# Health system performance for people with diabetes in 28 low- and middle-income countries: A cross-sectional study of nationally representative surveys

**DOI:** 10.1371/journal.pmed.1002751

**Published:** 2019-03-01

**Authors:** Jennifer Manne-Goehler, Pascal Geldsetzer, Kokou Agoudavi, Glennis Andall-Brereton, Krishna K. Aryal, Brice Wilfried Bicaba, Pascal Bovet, Garry Brian, Maria Dorobantu, Gladwell Gathecha, Mongal Singh Gurung, David Guwatudde, Mohamed Msaidie, Corine Houehanou, Dismand Houinato, Jutta Mari Adelin Jorgensen, Gibson B. Kagaruki, Khem B. Karki, Demetre Labadarios, Joao S. Martins, Mary T. Mayige, Roy Wong McClure, Omar Mwalim, Joseph Kibachio Mwangi, Bolormaa Norov, Sarah Quesnel-Crooks, Bahendeka K. Silver, Lela Sturua, Lindiwe Tsabedze, Chea Stanford Wesseh, Andrew Stokes, Maja Marcus, Cara Ebert, Justine I. Davies, Sebastian Vollmer, Rifat Atun, Till W. Bärnighausen, Lindsay M. Jaacks

**Affiliations:** 1 Divison of Infectious Diseases, Massachusetts General Hospital, Harvard Medical School, Boston, Massachusetts, United States of America; 2 Department of Global Health and Population, Harvard T.H. Chan School of Public Health, Boston, Massachusetts, United States of America; 3 Togo Ministry of Health, Lome, Togo; 4 Non-Communicable Diseases, Caribbean Public Health Agency, Port of Spain, Trinidad and Tobago; 5 Nepal Health Research Council, Kathmandu, Nepal; 6 Direction de la Lutte Contre la Maladie, Ministère de la Santé, Ouagadougou, Burkina Faso; 7 Ministry of Health, Victoria, Seychelles; 8 Institute of Social and Preventive Medicine, Lausanne, Switzerland; 9 The Fred Hollows Foundation NZ, Auckland, New Zealand; 10 Cardiology Department, Emergency Hospital of Bucharest, Bucharest, Romania; 11 Division of Non-Communicable Diseases, Kenya Ministry of Health, Nairobi, Kenya; 12 Health Research and Epidemiology Unit, Ministry of Health, Thimphu, Bhutan; 13 Department of Epidemiology and Biostatistics, School of Public Health, Makerere University, Kampala, Uganda; 14 Comoros Ministry of Health, Solidarity, Social Cohesion and Gender, Moroni, Comoros; 15 Laboratory of Epidemiology of Chronic and Neurological Diseases, Faculty of Health Sciences, University of Abomey-Calavi, Cotonou, Benin; 16 Partners in Health, Boston, Massachusetts, United States of America; 17 National Institute for Medical Research, Dar es Salaam, Tanzania; 18 Faculty of Medicine and Health Sciences, Stellenbosch University, Stellenbosch, South Africa; 19 Faculty of Medicine and Health Sciences, National University of East Timor, Dili, Timor-Leste; 20 Office of Epidemiology and Surveillance, Caja Costarricense de Seguro Social, San Jose, Costa Rica; 21 Zanzibar Ministry of Health, Mnazi Mmoja, Zanzibar; 22 National Center for Public Health, Ulaanbaatar, Mongolia; 23 St. Francis Hospital, Kampala, Uganda; 24 Non-Communicable Disease Department, National Center for Disease Control and Public Health, Tbilisi, Georgia; 25 Swaziland Ministry of Health, Mbabane, Swaziland; 26 Liberia Ministry of Health, Monrovia, Liberia; 27 Boston University Center for Global Health and Development, Boston, Massachusetts, United States of America; 28 Department of Economics and Centre for Modern Indian Studies, University of Göttingen, Göttingen, Germany; 29 MRC/Wits Rural Public Health and Health Transitions Research Unit, School of Public Health, University of the Witwatersrand, Johannesburg, South Africa; 30 Institute for Applied Health Research, University of Birmingham, Birmingham, United Kingdom; 31 Africa Health Research Institute, Somkhele, South Africa; 32 Institute of Public Health, Heidelberg University, Heidelberg, Germany; 33 Public Health Foundation of India, New Delhi, India; University of Cambridge, UNITED KINGDOM

## Abstract

**Background:**

The prevalence of diabetes is increasing rapidly in low- and middle-income countries (LMICs), urgently requiring detailed evidence to guide the response of health systems to this epidemic. In an effort to understand at what step in the diabetes care continuum individuals are lost to care, and how this varies between countries and population groups, this study examined health system performance for diabetes among adults in 28 LMICs using a cascade of care approach.

**Methods and findings:**

We pooled individual participant data from nationally representative surveys done between 2008 and 2016 in 28 LMICs. Diabetes was defined as fasting plasma glucose ≥ 7.0 mmol/l (126 mg/dl), random plasma glucose ≥ 11.1 mmol/l (200 mg/dl), HbA1c ≥ 6.5%, or reporting to be taking medication for diabetes. Stages of the care cascade were as follows: tested, diagnosed, lifestyle advice and/or medication given (“treated”), and controlled (HbA1c < 8.0% or equivalent). We stratified cascades of care by country, geographic region, World Bank income group, and individual-level characteristics (age, sex, educational attainment, household wealth quintile, and body mass index [BMI]). We then used logistic regression models with country-level fixed effects to evaluate predictors of (1) testing, (2) treatment, and (3) control. The final sample included 847,413 adults in 28 LMICs (8 low income, 9 lower-middle income, 11 upper-middle income). Survey sample size ranged from 824 in Guyana to 750,451 in India. The prevalence of diabetes was 8.8% (95% CI: 8.2%–9.5%), and the prevalence of undiagnosed diabetes was 4.8% (95% CI: 4.5%–5.2%). Health system performance for management of diabetes showed large losses to care at the stage of being tested, and low rates of diabetes control. Total unmet need for diabetes care (defined as the sum of those not tested, tested but undiagnosed, diagnosed but untreated, and treated but with diabetes not controlled) was 77.0% (95% CI: 74.9%–78.9%). Performance along the care cascade was significantly better in upper-middle income countries, but across all World Bank income groups, only half of participants with diabetes who were tested achieved diabetes control. Greater age, educational attainment, and BMI were associated with higher odds of being tested, being treated, and achieving control. The limitations of this study included the use of a single glucose measurement to assess diabetes, differences in the approach to wealth measurement across surveys, and variation in the date of the surveys.

**Conclusions:**

The study uncovered poor management of diabetes along the care cascade, indicating large unmet need for diabetes care across 28 LMICs. Performance across the care cascade varied by World Bank income group and individual-level characteristics, particularly age, educational attainment, and BMI. This policy-relevant analysis can inform country-specific interventions and offers a baseline by which future progress can be measured.

## Abstract 2

Using individual participant data from a large cohort study, Jennifer Manne-Goehler and colleagues reveal poor healthcare provision for diabetes patients in 28 LMICs.

## Author summary

**Why was this study done?:**

- Diabetes is a growing problem in low-income and middle-income countries, which already carry about 75% of the global diabetes burden, but there is limited evidence to guide policymaking in these settings.
- Prior studies have analyzed the cascade of care for diabetes in single-country or subnational contexts, but to our knowledge there have been no prior multi-country analyses on this subject.
- This study analyzes nationally representative data regarding health system performance for people who have diabetes, as defined by biological criteria, across a spectrum of levels of economic development.

**What did the researchers do and find?:**

- We harmonized nationally representative population-based survey data from 28 low-income and middle-income countries in 4 geographic regions, and used these data to construct the diabetes care cascade stratified by individual factors and national income.
- In this sample of over 800,000 adults, the overall prevalence of diabetes was 8.8% (95% CI: 8.2%–9.5%), of which 4.8% (95% CI: 4.5%–5.2%) was undiagnosed.
- In this study, we found that health system performance for diabetes showed large losses to care at the stage of detection (63.4% [95% CI: 56.7%–69.6%]) and low rates of diabetes control (22.8% [95% CI: 20.9%–24.9%]).
- Total unmet need for diabetes care (defined as the sum of undetected, detected but undiagnosed, diagnosed but untreated, and treated but not controlled diabetes) was 77.0% (95% CI: 74.9%–78.9%).
- Performance along the care cascade was better in upper-middle-income countries, but across all World Bank income groups, only 16%–25% of those with diabetes achieved diabetes control.
- Increasing age, educational attainment, and overweight or obesity were associated with higher odds of being tested, treated, and achieving control.

**What do these findings mean?:**

- There is a large unmet need for diabetes care that reflects the need for a greater investment of health resources to strengthen systems of care for this disease.
- Given that diabetes is considered a tracer condition for examining health systems, these findings also indicate that many countries face challenges in achieving universal health coverage.

## Introduction

Increases in life expectancy and epidemiological transition in low- and middle-income countries (LMICs) has led to a rapid rise in the burden of non-communicable diseases (NCDs), such as diabetes and cardiovascular diseases [1–4]. Health systems in resource-limited contexts, typically designed to provide services for maternal and child health and communicable diseases, now also need to respond to the burgeoning demand for care for chronic cardiometabolic diseases [5]. There is a need for robust evidence on current health system performance for management of diabetes in order to (1) design targeted policies and programs to improve patient outcomes and (2) provide a benchmark against which to compare future performance. To date, analyses on management of diabetes have been limited by scant empirical data on prevalence and utilization of health services [6], in particular at the level of the individual [7].

One analytic approach to assessing health system performance is the construction of the cascade of care [8]. The cascade of care approach has been widely used to monitor performance and to examine response to the HIV epidemic and progress toward coverage goals for populations affected by HIV [9,10]. This method uses a quantitative depiction of the stepwise care system for the population affected by a disease of interest, typically involving testing, diagnosis, treatment initiation, adherence to treatment, and effective control. The primary strength of cascade of care analysis is the ability to identify where in the continuum of care the greatest losses to care occur [9]. This in turn can help facilitate effective, evidence-based targeting of programs and policies to address these gaps. This analytic approach is being increasingly applied at the national level to examine management of chronic diseases such as hypertension and diabetes [11–15].

The aims of this analysis—which examines to our knowledge the largest set of nationally representative surveys in LMICs to date—were (1) to provide the first estimates of health system performance for management of diabetes using a cascade of care approach and (2) to assess predictors of diabetes testing, treatment, and control in these resource-limited settings. In this study, we first utilize data from population-based surveys in 28 countries to construct cascades of care for diabetes. We then use individual-level data to evaluate predictors of reaching particular stages in the cascade, namely testing of glucose biomarkers, treatment of diabetes, and glycemic control.

## Methods

### Data sources

We did a pooled cross-sectional analysis of individual-level data from 28 nationally representative population-based surveys [13,16–21]. The requirements for inclusion of a country survey in this study were as follows: (1) The survey was conducted during or after 2008 (in cases where 2 or more surveys were available for a particular country, the most recent survey was used); (2) the survey data were made available at the individual level; (3) the survey contained a biomarker for diabetes (either a glucose measurement or HbA1c); (4) the survey was conducted in an upper-middle, lower-middle, or low-income country according to the World Bank at the time the survey was conducted; (5) the survey was nationally representative; (6) the survey had a response rate ≥ 50%; (7) the survey included a suite of questions that assessed access to a core and comparable group of health services for diagnosis, preventive counseling, and treatment of diabetes.

We first identified all countries in which a World Health Organization (WHO) Stepwise Approach to Surveillance (STEPS) survey had been conducted in a year when the country fell into an eligible World Bank income category of low income or middle income. The STEPS survey is a standardized instrument for collecting and disseminating data about NCD risk factors in adults living in WHO member countries [22]. We systematically requested each eligible STEPS survey from a list of these surveys that WHO maintains on its website [23]. The research team contacted the responsible person for each survey, based on the information provided on this website. If the contact information was outdated or unavailable, the authors relied on publications utilizing STEPS data and electronic searches of the survey or contact name. For the Caribbean region, country involvement was facilitated by the Caribbean Public Health Agency (CARPHA).

Of the 109 STEPS surveys conducted (105 of which are listed on the WHO website), 52 met our inclusion criteria (S1 Appendix). Of these, 18 had a contact person who did not respond to our request for data, 2 had a contact person who declined our request for data, 5 did not have valid contact information, and 9 were not yet shared at the time of locking the data for analysis (1 April 2018). This analysis included a total of 18 eligible STEPS surveys for the following countries: Benin, Bhutan, Burkina Faso, Comoros, Costa Rica, Georgia, Guyana, Kenya, Liberia, Mongolia, Nepal, Saint Vincent and the Grenadines, Seychelles, Swaziland, Tanzania, Timor-Leste, Togo, and Uganda.

Of the remaining LMICs without an eligible STEPS survey listed on the WHO website or those listed but without valid contact information or for which the contact person declined our request for data (97 countries total), we did a systematic Google search and identified 35 potentially eligible non-STEPS surveys (S2 Appendix). Of these, 7 had a contact person who did not respond to our request for data, 4 had a contact person who declined our request for data, 3 did not have valid contact information, and 12 did not have a biomarker for diabetes. Since the Google search was completed (1 January 2018), an additional eligible survey was made public (the Indian National Family Health Survey [NFHS]). Thus, we included data from a total of 10 countries with an eligible non-STEPS survey: the 2011 Bangladesh Demographic and Health Survey (DHS), the 2009–2010 Chile National Health Survey, the 2009 China Health and Nutrition Survey, the 2009 Fiji Eye Health Survey, the 2015–2016 Indian NFHS, the 2014–2015 Indonesian Family Life Survey, the 2009–2012 Mexico Family Life Survey, the 2013 Namibia DHS, the 2015–2016 Study for the Evaluation of Prevalence of Hypertension and Cardiovascular Risk in Romania III, and the 2012 South African National Health and Nutrition Examination Survey.

Most of the surveys used 2-stage cluster random sampling designs (S3 Appendix). All surveys measured height and weight, which we used to calculate body mass index (BMI). BMI was defined as weight (measured in kilograms) divided by height (measured in meters) squared, and classified as underweight (<18.5 kg/m^2^), normal weight (18.5 to <25.0 kg/m^2^), overweight (25.0 to <30.0 kg/m^2^), or obese (≥30.0 kg/m^2^) [24]. Because the survey in Bangladesh measured height and weight only among women, it was excluded from those regressions that included BMI as a predictor variable.

The surveys assessed different indicators of household wealth including continuous income, income categories, income quintiles, an asset index, or a combination of these (S4 Appendix). In an effort to leverage all available information on wealth and create a comparable measure across surveys, we constructed household wealth quintiles for each survey. Because Burkina Faso, Chile, Costa Rica, Fiji, and Seychelles did not assess any of these comparable wealth indicators, they were excluded from those regressions that included household wealth quintile as a predictor variable. Educational attainment was classified as no formal schooling, primary school (grade 6), or secondary school (grade 7 to grade 12) or above. We used local education categorical variables when available—or, if not available, years of education completed (a continuous variable)—to classify all participants according to these categories. This study was deemed exempt from institutional ethics approval.

### Diabetes biomarkers

A point-of-care fasting capillary glucose measurement was the diabetes biomarker for 20 of the 28 countries (S5 Appendix). Plasma equivalents were provided by all but 7 of these surveys; for these 7, we multiplied the capillary glucose by 1.11 so that all values were plasma equivalents. This adjustment was made based on published guidelines and evidence that has shown that capillary glucose often underestimates plasma glucose levels [2,25,26]. A laboratory-based measurement of fasting plasma glucose was the diabetes biomarker for 4 of the 28 countries. For the remaining 4 countries, only glycated hemoglobin A1c (HbA1c) was available (Fiji, Indonesia, Mexico, and South Africa).

### Definitions of diabetes

Diabetes was defined based on the present WHO diagnostic criteria as any of the following: a fasting plasma glucose of 7.0 mmol/l (126 mg/dl) or higher; a random plasma glucose of 11.1 mmol/l (200 mg/dl) or higher; or, in the case of Fiji, Indonesia, Mexico, and South Africa, an HbA1c measurement of 6.5% or higher [27]. Participants with missing data on fasting status were assumed to be fasting as they were told to fast as part of all survey protocols, except in India, where participants were not instructed to fast. We performed a sensitivity analysis in which we assumed all participants who were missing data on fasting status to be non-fasting.

Individuals reporting use of drugs for diabetes were also classified as having diabetes, irrespective of their biomarker values. Respondents who self-reported a diagnosis of diabetes but were not on drug treatment and did not meet the biomarker diagnostic criteria were not classified as having diabetes.

### Constructing the diabetes care cascade

The stages of the care cascade were testing, diagnosis, lifestyle advice and/or medication given (“treatment”), and control (HbA1c < 8.0% or glucose < 10.1 mmol/l), described in detail below. A table of the generic questions used to construct the cascade is provided in S6 Appendix.

#### Ever had a blood glucose test (“testing”)

The questionnaires used in Bangladesh, China, Fiji, India, Indonesia, Mexico, and Romania did not specifically query whether or not respondents had ever had a blood glucose test, and therefore this step was not included in the cascades for these countries. All other surveys explicitly asked whether or not the respondent had ever had a blood glucose test.

#### Ever diagnosed with diabetes (“diagnosis”)

With the exceptions of Bangladesh, China, Fiji, India, Indonesia, Mexico, and Romania (which did not ask whether or not respondents had ever had a blood glucose test, and thus their value for the ever diagnosed with diabetes variable was as reported), if a respondent said that s/he never had a blood glucose test, the value for this variable (ever diagnosed with diabetes) was set to “no.” If a respondent said that s/he did have a blood glucose test, the value for this variable (ever diagnosed) was analyzed as reported (“yes” or “no”).

#### Lifestyle advice

The questionnaires in Bangladesh, Georgia, India, Mexico, Romania, and South Africa did not include any lifestyle advice questions. The questionnaire in Chile asked about treatment without medication (exercise, diet, losing weight). Similarly, the questionnaire in Fiji asked about treatment without medication (diet and exercise, but not losing weight). The questionnaire in China did not ask about exercise, but did ask about losing weight and diet. The questionnaire in Namibia did not ask about diet, but did ask about exercise and losing weight. Participants were assigned a value of “yes” if they responded “yes” to receiving advice about losing weight, exercising, or following a special diet (including a low-fat diet), either specific to their diabetes or generally. If a respondent said that s/he had never been diagnosed with diabetes, the value for this variable (received lifestyle advice) was “no.”

#### Currently taking diabetes medications

For 3 countries (India, Namibia, and Uganda), the question regarding diabetes medications did not specify whether or not it included both oral medications and insulin. For all other countries, we were able to distinguish between oral medications and insulin because separate questions were asked (“yes” or “no” to currently taking drugs [medication] or insulin, or a self-reported medication list from which insulin use was determined). If a respondent said that s/he had never been diagnosed with diabetes, the value for this variable (currently taking diabetes medications) was “no.”

Using these variables, we constructed a diabetes care cascade for each country. This cascade, created using individual participant data, shows the percent of the total population with diabetes that self-reported reaching subsequent stages in the care process, conditional on having reached the previous stage. In other words, the denominator is fixed as all participants with diabetes. The numerator is the subset of participants with diabetes who achieved the given stage of the cascade and all previous stages of the cascade.

The first stage in our cascade was ever had a blood glucose test (prior to the STEPS or other survey in which the diagnosis was made for the purposes of this study) as an indicator of diabetes testing. Among those who had been tested, we then quantified the percentage of all patients with diabetes who reported having been diagnosed with diabetes by any healthcare provider as a measure of awareness of diagnosis. Third, among those who had been tested and diagnosed, we calculated the percentage of the population who received any advice regarding lifestyle modification and the percentage who received oral medication or insulin for diabetes control. These 2 indicators were then used in a composite endpoint called “treatment.” Finally, among those receiving oral medication or insulin and/or lifestyle advice, we determined whether or not their diabetes was in control as the final stage of the cascade. For countries with plasma glucose measurements, “control” was defined as plasma glucose < 10.1 mmol/l, and for countries with HbA1c (Fiji, Indonesia, Mexico, and South Africa), “control” was defined as HbA1c < 8.0%.

Total unmet need for diabetes care was defined as the sum of those not tested, tested but undiagnosed, diagnosed but untreated, and treated but with diabetes uncontrolled. Individuals with controlled diabetes were not included in the definition of unmet need for care.

In addition to calculating a care cascade for each country, we also calculated an overall care cascade for the total population included in the study as well as by geographic region and World Bank income group. Country regional and World Bank income group classifications are provided in S3 Appendix. Finally, we also calculated care cascades by individual-level characteristics (age, sex, BMI status, educational attainment, and household wealth quintile). Differences in cascade completion across these variables were evaluated for statistical significance using a Pearson (Rao–Scott correction F-statistic) chi-squared test.

### Statistical analysis

All analyses were done in Stata v. 14.2 (StataCorp, College Station, Texas, US) with point estimates and variance taking into account the survey design. When available, sample weights corresponding to the biomarker examination (e.g., subsample weights) were used. However, these sample weights did not account for non-response. When sample weights were missing for an observation within a country, the mean sample weight for all observations in that country was assigned. For estimates (including regression analyses) that included more than 1 country, sample weights were rescaled by the survey’s sample size such that all countries contributed equally to the overall estimates. This choice was made because the analysis unit of interest in this study was a national health system. Despite accounting for a large proportion of the total sample size, the data from India therefore do not influence the summary statistics and regression estimates shown in this paper more than the data from any of the other countries. A subpopulation command was used to specify non-pregnant adults 15 years or older for all estimates.

Logistic regression models with country-level fixed effects and standard errors adjusted for clustering at the country level were used to evaluate predictors of 3 endpoints of cascade completion: (1) tested, (2) treated, and (3) controlled. Predictors included age (15–34 years, 35–44 years, 45–54 years, or ≥55 years), sex, educational attainment (no formal schooling, primary school, or secondary school or above), household wealth quintile, and BMI (underweight, normal weight, overweight, or obese). Overall, less than 20% of participants with diabetes were missing data for these predictor variables, and so we conducted a complete-case analysis. The number and proportion of participants with diabetes missing data by country is provided in S7 Appendix. This study did not have a pre-established analysis plan or published protocol.

## Results

Our final sample included 847,413 individual participants in 28 LMICs (Table 1). The overall prevalence of diabetes across these 28 countries when using equal weighting for each country was 8.8% (95% CI: 8.2%–9.5%). The country-level prevalence across these surveys ranged from a low of 1.4% (95% CI: 1.0%–2.1%) in Uganda to a high of 43.2% (95% CI: 39.7%–46.8%) in Fiji (S8 Appendix). When stratified by geographic region, we found that the prevalence was lowest in sub-Saharan Africa (5.3% [95% CI: 4.2%–6.7%]) and highest in Latin America and the Caribbean (15.3% [95% CI: 14.1%–16.6%]). The prevalence of diabetes was also higher among countries in higher World Bank income groups (low income, 4.2% [95% CI: 3.5%–5.0%]; lower-middle income, 5.1% [95% CI: 4.7%–5.5%]; upper-middle income, 15.3% [95% CI: 14.3%–16.4%]). Overall, the prevalence of undiagnosed diabetes was 4.8% (95% CI: 4.5%–5.2%). Among the population with diabetes, 58.2% were female, 45.7% were ≥55 years old, 44.1% had secondary school education or above, and 39.2% had obesity (Table 2).

**Table 1:** Summary of population-based surveys conducted in 28 low- and middle-income countries between 2008 and 2016 and country-level characteristics.

Country	Year	Response rate (%)*	Sample size^†^	Mean age (years)^‡^	Female (%)^‡^	World Bank income group^§^	Health expenditures per capita^#^
**Total**			847,413	41.1	53.3		
Bangladesh	2011	95.0	7,305	51.3	49.7	Lower-middle	31
Benin	2008	99.0	3,521	43.3	51.0	Low	38
Bhutan	2014	96.9	2,674	40.5	61.1	Lower-middle	89
Burkina Faso	2013	97.8	3,945	38.9	50.7	Low	35
Chile	2009–2010	85.0	4,874	46.4	60.0	Upper-middle	1,137
China	2009	88.0 (2006)	8,707	50.4	52.6	Upper-middle	420
Comoros	2011	96.5	2,295	41.7	73.9	Low	57
Costa Rica	2010	87.8	2,592	49.8	72.8	Upper-middle	970
Fiji	2009	80.0	1,344	55.5	57.1	Upper-middle	204
Georgia	2016	75.7	3,160	49.0	72.0	Lower-middle	303
Guyana	2016	66.7	824	41.6	62.7	Upper-middle	222
India	2015–2016	96.0	750,451	30.5	85.6	Lower-middle	75
Indonesia	2014–2015	83.0	6,483	43.8	54.6	Lower-middle	99
Kenya	2015	95.0	3,974	38.1	59.5	Lower-middle	78
Liberia	2011	87.1	2,183	38.6	56.7	Low	46
Mexico	2009–2012	~90	9,037	48.2	54.7	Upper-middle	677
Mongolia	2009	95.0	1,572	39.2	40.0	Lower-middle	195
Namibia	2013	96.9	3,244	47.0	58.1	Upper-middle	499
Nepal	2013	98.6	3,742	41.1	68.0	Low	40
Romania	2015–2016	69.1	1,969	48.5	52.5	Upper-middle	557
Seychelles	2013	73.0	1,240	45.7	57.2	Upper-middle	494
South Africa	2012	92.6	4,615	40.8	64.2	Upper-middle	570
Saint Vincent and the Grenadines	2013	67.8	987	43.7	60.4	Upper-middle	575
Swaziland	2014	81.8	2,809	37.1	64.0	Lower-middle	248
Tanzania	2012	94.7	4,724	41.8	52.2	Low	52
Timor-Leste	2014	96.3	2,334	41.3	58.1	Lower-middle	57
Togo	2010	91.0	3,400	34.8	50.6	Low	34
Uganda	2014	99.0	3,408	35.8	58.4	Low	52

*Response rate for questionnaire/interview.

^†^Number of participants with non-missing diabetes biomarker, non-pregnant, and aged ≥15 years. Unweighted.

^‡^The value for the total row accounts for sampling design, with survey weights giving each country the same weight. Country-level values are unweighted.

^§^Country classification at time of survey according to World Bank gross national income per capita in US dollars (Atlas methodology) (https://datahelpdesk.worldbank.org/knowledgebase/articles/906519-world-bank-country-and-lending-groups).

^#^Health expenditures per capita in 2014 (current US dollars) from World Bank (https://data.worldbank.org/indicator/).

^¶^Response rate for the 2006 wave of the survey (the most recent wave for which a response rate was published).

**Table 2:** Baseline characteristics of participants with diabetes (*n* = 40,701) in population-based surveys conducted in 28 low- and middle-income countries between 2008 and 2016.

Characteristic	Weighted percent (unweighted *n*)*
Sex	
Male	41.8 (9,708)
Female	58.2 (30,993)
Missing	0
Age	
15–34 years	9.7 (13,340)
35–44 years	16.1 (11,702)
45–54 years	28.4 (10,824)
≥55 years	45.7 (4,835)
Missing	0
Educational attainment	
No formal schooling	17.6 (9,826)
Primary school	38.2 (7,575)
Secondary school or above	44.1 (22,773)
Missing	527
Household wealth quintile	
1	17.7 (4,675)
2	20.4 (5,503)
3	16.2 (6,676)
4	22.0 (8,772)
5	23.6 (10,961)
Missing	4,114
Body mass index classification	
Underweight	2.9 (3,111)
Normal weight	25.8 (14,007)
Overweight	32.0 (9,824)
Obese	39.2 (6,483)
Missing	7,276

*Percent accounts for sampling design, with survey weights giving each country the same weight. Unweighted *n*. For “Missing,” value is unweighted *n*.

**Fig 1:**
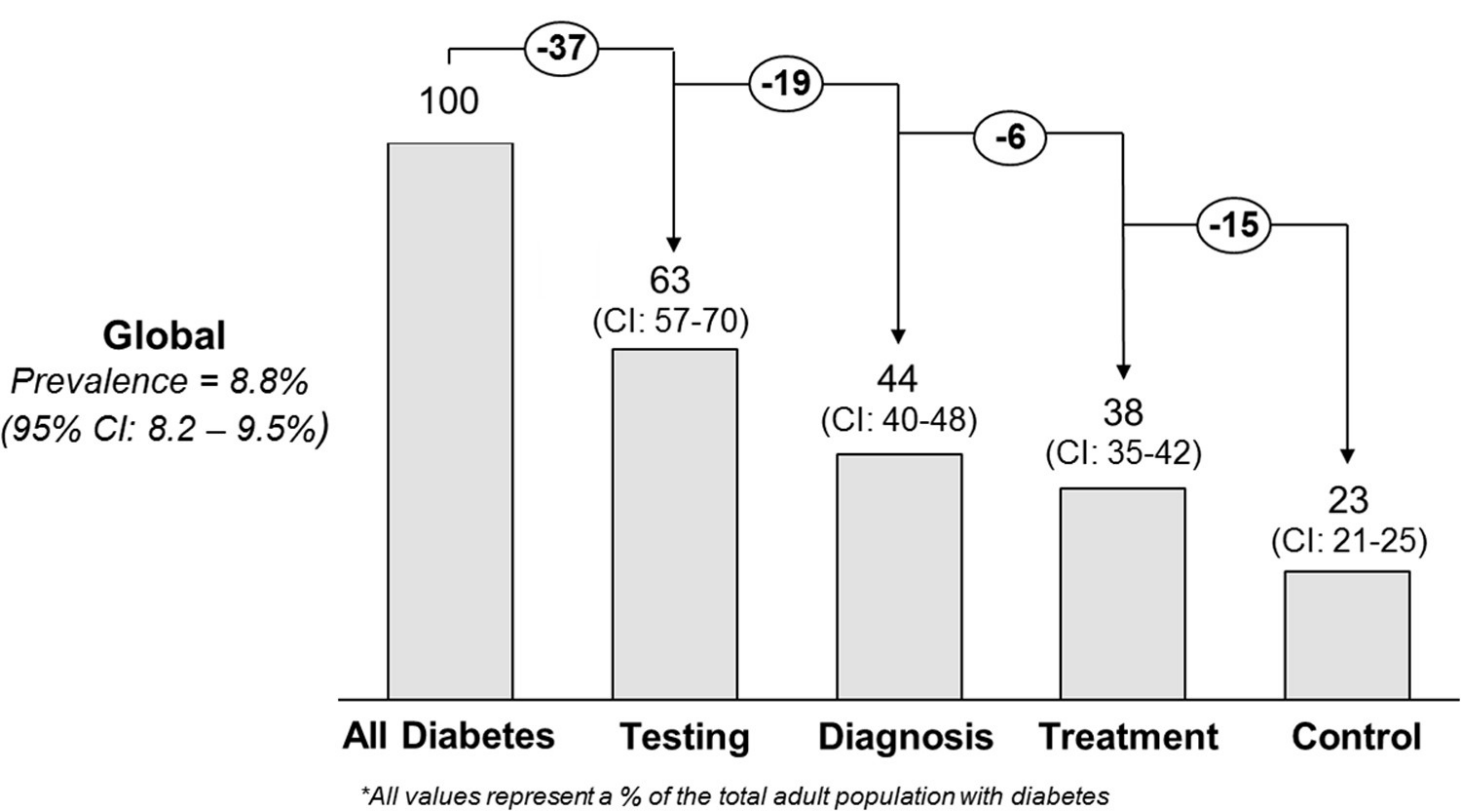
The global diabetes cascade of care in population-based surveys conducted in 28 low- and middle-income countries between 2008 and 2016.

The overall cascade of care analysis with all countries weighted equally revealed the largest loss to care at the stage of testing: only 63.4% (95% CI: 56.7%–69.6%) of those with diabetes had ever been tested with a blood glucose measurement (Fig 1). There was a more modest loss to care at the stage of being told about this diagnosis, with 44.3% (95% CI: 40.2%–48.4%) of those with diabetes reporting that they were aware of their diagnosis. Furthermore, only 38.4% (95% CI: 35.2%–41.7%) of those with diabetes received treatment with lifestyle modification advice and/or medications for their diabetes. Finally, 22.8% (95% CI: 20.9%–24.9%) of those with diabetes had achieved control of their disease. In sensitivity analyses, assuming all participants who were missing data on fasting status were non-fasting or excluding India did not substantively impact the findings for the prevalence or cascade performance (S9 Appendix).

**Fig 2:**
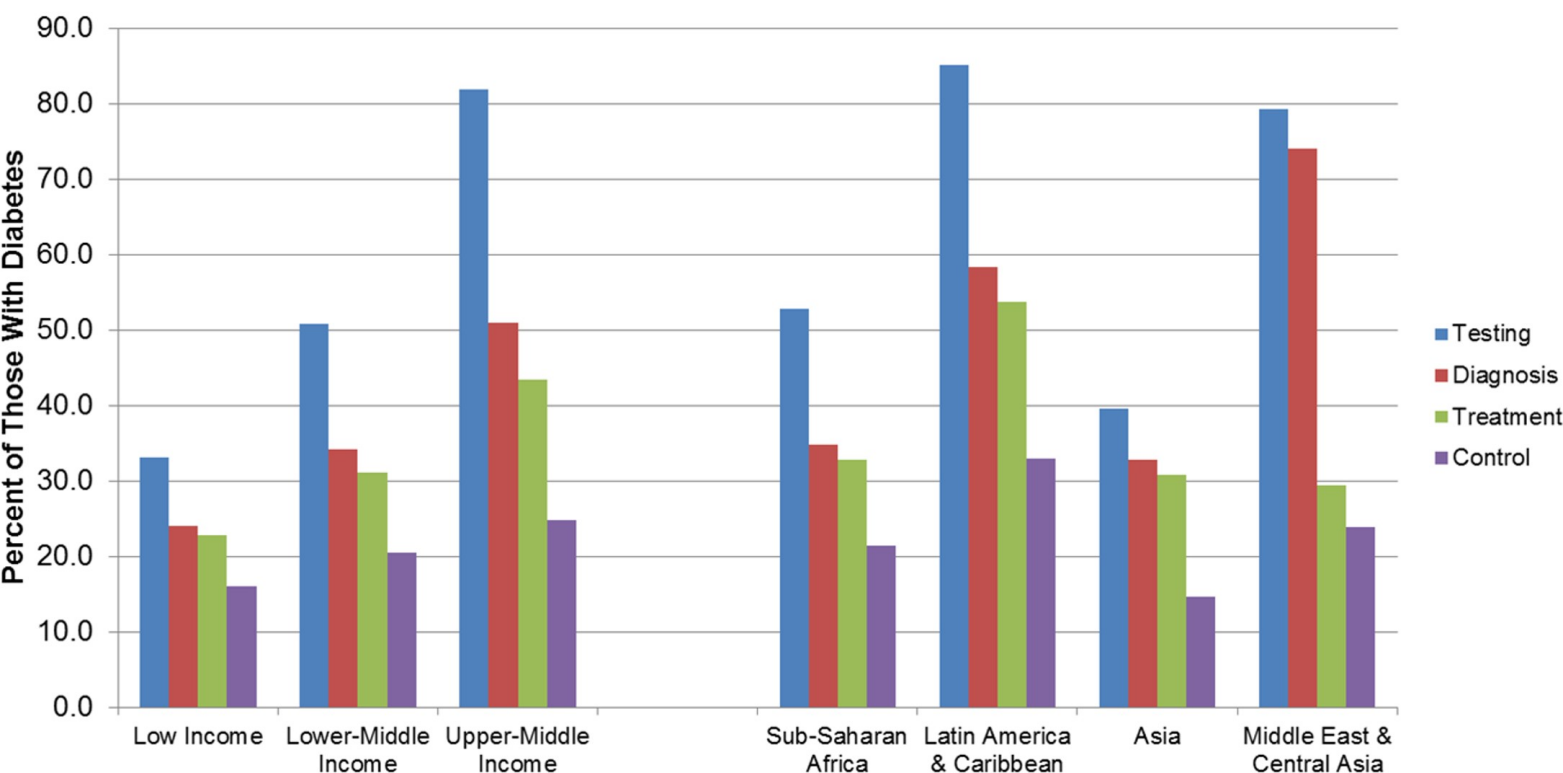
The diabetes cascade of care by world bank income group and geographic region in population-based surveys conducted in 28 low- and middle-income countries between 2008 and 2016. “Asia” is South and Southeast Asia.

When stratified by region, performance of diabetes management across the care cascade (hereafter performance) was substantially better in Latin America and the Caribbean and the Middle East and Central Asia, as compared to performance in either sub-Saharan Africa or South and Southeast Asia (*p <* 0.001 for treatment and control; Fig 2). Rates of treatment were higher in countries in higher World Bank income groups (*p <* 0.001 for treatment and *p <* 0.001 for control; Fig 2). There was substantial variation in the relative percent loss to care between steps of the care cascades across countries, though the overall picture was largely similar in terms of greatest losses at testing, and smallest losses between diagnosis and treatment (S10 Appendix). Costa Rica stood out as having among the lowest losses to care at each stage of the cascade. Tanzania and Benin also had relatively low losses to care between testing and diagnosis, but substantially larger losses to care between treatment and control.

**Fig 3:**
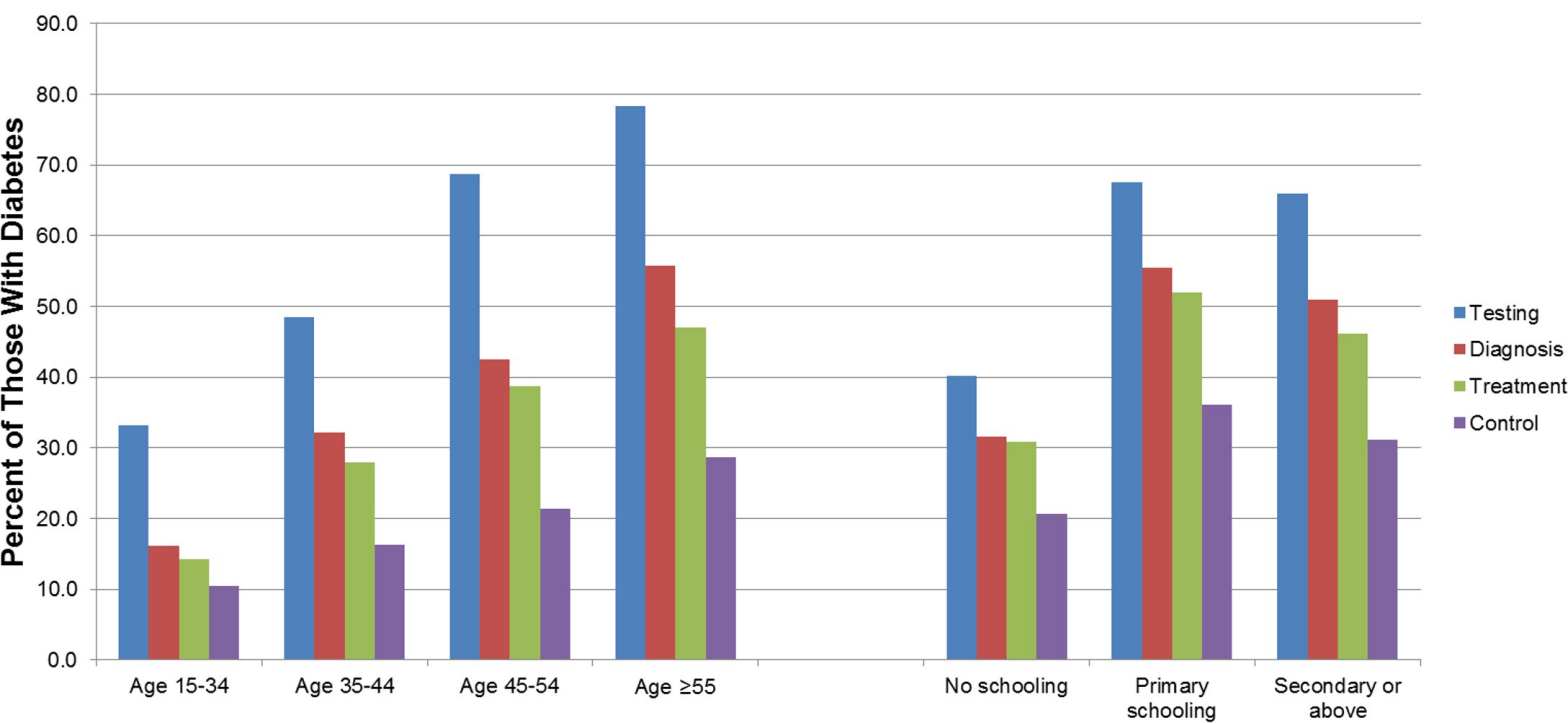
The diabetes cascade of care by age group and educational attainment in population-based surveys conducted in 28 low- and middle-income countries between 2008 and 2016.

When the cascade of care was stratified by sociodemographic characteristics, there was little difference between men and women, particularly with respect to control (*p =* 0.22 for treatment and *p =* 0.56 for control), but there were large differences in performance across 10-year age groups (*p <* 0.001 for both treatment and control) (Fig 3). Performance across the care cascade was poorest in the age group 15–34 years and higher in older age groups across all stages of the cascade, with the best performance in the oldest age group (≥55 years old). Those who had obesity were more likely to be treated and to have their diabetes controlled than those who were underweight (*p <* 0.001 for treatment and *p <* 0.001 for control). With respect to educational attainment (*p =* 0.001 for treatment and *p =* 0.043 for control) and household wealth quintile (*p =* 0.031 for treatment and *p =* 0.690 for control), there was no clear pattern across groups.

With respect to treatment, we found that more people reported treatment with medication (35.5% [95% CI: 32.6%–38.6%]) than being advised regarding lifestyle modifications to improve their diabetes control (18.9% [95% CI: 15.9%–22.3%]). Moreover, the majority of those who reported treatment with medications also reported having received lifestyle advice (64.5% [95% CI: 58.5%–70.1%]). With respect to medications, 7.9% (95% CI: 6.8%–9.1%) of those with diabetes reported use of insulin (estimate excludes India, Namibia, and Uganda, for which information specific to insulin use was not collected).

The results of regression models showed statistically significant gradients of age, educational attainment, and BMI status with the odds of testing, treatment, and control (Table 3). Univariable results for each of these predictors are provided in S11 Appendix.

**Table 3:** Multivariable logistic regression analyses assessing the relationship between sociodemographic characteristics and testing, treatment, and control of diabetes in population-based surveys conducted in 22 low- and middle-income countries between 2008 and 2016.*

Covariate	OR (95% CI)		
	Testing^†^	Treatment	Control
Sex			
Male	Ref	Ref	Ref
Female	1.19 (0.91–1.56)	1.10 (0.88–1.37)	1.10 (0.83–1.45)
Age			
15–34 years	0.54 (0.46–0.62)	0.32 (0.23–0.44)	0.42 (0.25–0.69)
35–44 years	Ref	Ref	Ref
45–54 years	1.57 (1.45–1.71)	2.57 (1.96–3.37)	2.52 (1.63–3.90)
≥55 years	2.06 (1.83–2.33)	4.43 (3.20–6.14)	4.77 (2.82–8.06)
Educational attainment			
No formal schooling	Ref	Ref	Ref
Primary school	1.60 (1.24–2.07)	1.64 (1.28–2.09)	1.25 (0.93–1.67)
Secondary school or above	2.84 (2.02–3.99)	2.18 (1.62–2.94)	2.11 (1.45–3.07)
Household wealth quintile			
1	Ref	Ref	Ref
2	1.03 (0.84–1.26)	0.92 (0.71–1.21)	0.97 (0.78–1.21)
3	1.17 (0.88–1.56)	0.86 (0.65–1.14)	0.87 (0.62–1.21)
4	1.32 (0.95–1.83)	1.11 (0.81–1.53)	1.02 (0.73–1.43)
5	1.93 (1.25–2.98)	1.18 (0.76–1.81)	1.04 (0.64–1.70)
Body mass index classification			
Underweight	0.69 (0.59–0.81)	0.47 (0.27–0.81)	0.41 (0.25–0.66)
Normal weight	Ref	Ref	Ref
Overweight	1.70 (1.41–2.05)	2.00 (1.42–2.82)	2.08 (1.63–2.66)
Obese	2.60 (2.10–3.22)	3.05 (2.18–4.28)	3.23 (2.40–4.33)

*Bangladesh, Burkina Faso, Chile, Costa Rica, Fiji, and Seychelles were dropped from multivariable prediction models due to a lack of data on body mass index (Bangladesh) or household wealth quintile (Burkina Faso, Chile, Costa Rica, Fiji, and Seychelles). Univariable prediction models for each of these predictors are presented in S11 Appendix.

^†^Additionally does not include China, India, Indonesia, Mexico, and Romania because the questionnaires used in these countries did not specifically query whether or not respondents had ever had a blood glucose test.

In the multivariable regression models, age ≥ 55 years was associated with an odds of testing 2.06 (95% CI: 1.83–2.33) times higher, an odds of treatment 4.43 (95% CI: 3.20–6.14) times higher, and an odds of control 4.77 (95% CI: 2.82–8.06) times higher than age 15–34 years. Moreover, having a secondary school education or above was associated with an odds of testing 2.84 (95% CI: 2.02–3.99) times higher, an odds of treatment 2.18 (95% CI: 1.62–2.94) times higher, and an odds of control 2.11 (95% CI: 1.45–3.07) times higher than having no formal schooling. Those who had obesity had 2.60 (95% CI: 2.10–3.22) times higher odds of testing, 3.05 (95% CI: 2.18–4.28) times higher odds of treatment, and 3.23 (95% CI: 2.40–4.33) times higher odds of control compared to those who were normal weight. There were no statistically significant relationships between sex or household wealth quintile and any of the 3 outcomes, except that those with the greatest wealth had 1.93 (95% CI: 1.25–2.98) times higher odds of testing than those with the lowest wealth.

## Discussion

To the best of our knowledge, this study provides the first empirical analysis of health system performance for management of diabetes across LMICs in multiple geographic regions. Overall, we found that health system performance for diabetes management in these settings can generally be characterized by large losses to care at the stage of diabetes testing and only moderate rates of diabetes control despite using a lenient definition of glycemic control. These losses might be due to demand-side factors such as lack of patient awareness and engagement, inability to afford care, or sociocultural barriers, or supply-side factors such as lack of services, poor responsiveness of the services provided, or geographic inaccessibility [5].

Total unmet need for diabetes care (defined as the sum of those not tested, tested but undiagnosed, diagnosed but untreated, and treated but with diabetes not controlled) was high, at 77%. We also identified important variation in health system performance in management of diabetes by region, World Bank income group, and individual-level sociodemographic factors. Specifically, we found that individuals with diabetes who live in upper-middle-income countries are more likely to be tested, diagnosed, and treated for their diabetes than those in low-income and lower-middle-income countries, but that in any given World Bank income group, only 16%–25% of those with diabetes ultimately achieve control. These findings suggest that countries with greater wealth and, in turn, more health systems resources are effectively reaching and engaging more people with diabetes, but that there are similar difficulties for health systems across all income groups in translating services delivered into effective disease control.

Our finding of large losses to care at the stage of diabetes testing represents a challenge for the health systems examined, but with global implications due to a lack of clear consensus about the optimal approach to screening adults for diabetes [28]. Several studies conducted in high-income countries have failed to find a mortality benefit associated with diabetes screening, and leading medical organizations in these settings offer inconsistent recommendations regarding at what age, with what risk profiles, and with what frequency to screen for diabetes [29,30]. While we find large losses to care at this first stage of screening, this finding should be understood in the context of a lack of clear global guidelines to direct screening and screening activities in LMICs.

The results of this study have several important policy implications for health systems strengthening in the context of a growing global diabetes epidemic. First, we found that those individuals who were older and those who were overweight or obese had better performance across the diabetes care cascade, including greater rates of testing, diabetes service utilization, and disease control, as compared to younger or underweight individuals. This suggests that the health systems included in this analysis might be appropriately targeting resources to those at greatest risk of diabetes based on their age and comorbid overweight or obesity. It may also partially reflect a survivor bias in that we are only capturing healthier older adults with diabetes who have access to care. Our findings also indicate that certain subsets of the population appear to be “left behind” in terms of the reach of diabetes services, namely younger and underweight individuals who have diabetes. While there are limited data to guide screening for these lower-risk and relatively lower-prevalence subpopulations, there is a need to strengthen linkage to care for these individuals. Second, we found that the majority of adults who were diagnosed also reported receiving at least 1 form of treatment (either medications or lifestyle advice), and thus the proportion of those who were diagnosed and whose diabetes was in control was similar to the proportion of those who were treated and whose diabetes was in control (26.5% versus 22.8%). However, when we disaggregated the forms of treatment that were offered, we found that 10% of those treated were actually receiving only lifestyle modification advice (e.g., no medication), and, among those, only 18% achieved control. In contrast, 13% of the sample was treated with medications in the absence of lifestyle modification advice, and, among those, 58% achieved control. While we would not advocate for a strategy of medicating patients without also counseling them on lifestyle factors that contribute to their disease risk and progression, this evidence suggests that lifestyle advice alone is likely insufficient to achieve higher rates of glycemic control at a population level. Further research, including continued, frequent national monitoring of the diabetes care cascade, is needed to provide more nuanced insights into this observation and targets for intervention.

There are several published studies that have used a cascade of care approach to assess health system performance for diabetes and can serve as a benchmark for these findings. The first is a study by Ali and colleagues using data from the US National Health and Nutrition Examination Survey. These investigators found that 12% of US adults had diabetes and that among these, 72% had been diagnosed [11]. In the US context, those who were diagnosed had lower rates of glycemic control (64%) than those who were undiagnosed (77%) [11]. These rates of both diagnosis and glycemic control are similar to those in the upper-middle income countries included in our study, but much higher than those in the low- or lower-middle-income countries included in our study. This pattern is consistent with our finding that health systems in higher-resource settings have better cascade performance for diabetes, especially as relates to the reach of health services. However, while absolute rates of glycemic control were higher in the US and in the upper-middle-income countries in this study, Ali and colleagues also observed large losses between the stage of engagement in care, analogous to the “treated” stage in our analysis, and glycemic control in the US context. Though there are modest differences in the measurement of this stage in the cascade between these studies, we also found that the health systems of many LMICs struggle to translate service coverage into glycemic control, a key clinical and health systems metric for the diabetes epidemic. This common finding suggests that patient adherence and glycemic control are difficult to achieve and that this challenge is less closely linked to health systems resources. This finding also emphasizes the need for innovative, scalable, and locally acceptable approaches to influence human behavior in order to improve rates of glycemic control.

In addition, 3 recent studies have used the cascade of care approach to understand national-level health system performance for diabetes in resource-strapped settings. The first, from Seychelles, showed an age-standardized prevalence of diabetes of 9.6% in men and 9.1% in women in 2004 [16]. This study also confirmed that at this time, about 54% of people with diabetes were aware of this diagnosis, the vast majority of whom were treated (98%) but very few of whom had their diabetes controlled (21%) [16]. The second is a study that used nationally representative data from South Africa, also included in this pooled analysis, and found an age-standardized prevalence of diabetes of 10% [13]. Among participants with diabetes, 45% had not been screened, 15% had been screened but were not diagnosed, 2.3% had been diagnosed but had not been treated, and 18% had been treated but had not achieved glycemic control [13]. The third, a population-based study of both urban and rural residents in Malawi, found a prevalence of diabetes of 2%–3% and determined that 59% of people with diabetes were diagnosed, 62% of those who had been diagnosed were also treated, and 41% of those who had been treated had their diabetes in control [12]. Though these studies quantified losses in the cascade using conditional probabilities (e.g., their denominator changed with each stage of the cascade, whereas we used a fixed denominator of all participants with diabetes across all stages of the cascade), they ultimately uncovered comparable absolute rates of diagnosis and control to our study.

In addition to this literature, there have been several systematic reviews that further complement and reinforce aspects of the original research presented herein. For instance, a recent systematic review showed major gaps in guidelines for the management of diabetes in resource-limited health systems as compared to guidelines used in the health systems of high-income settings [31]. Specifically, this systematic review showed that only about 12% of diabetes guidelines from LMICs satisfied at least 4 of the Institute of Medicine’s standards, as opposed to 60% of the guidelines from high-income settings [31]. In a second systematic review of 93 studies—7 of which were conducted in LMICs—that assessed the effects of health systems factors, interventions, policies, or programs on diabetes awareness, treatment, control, and treatment adherence, the authors concluded that limited access to health services and medication was a leading health systems barrier [32]. However, the authors of this study reported that the heterogeneity in methods used across the studies included in the review prevented them from performing a meta-analysis [32]. Our study overcomes this barrier by harmonizing data across 28 LMICs and conducting an original analysis using the resulting multi-country dataset.

Our findings also provide specific, positive examples of excellent cascade performance, especially in the case of Costa Rica. Costa Rica has successfully implemented universal health coverage with unified financing, positioning primary healthcare at the center of its health services network and emphasizing care for leading cardiovascular risk factors including diabetes [33]. Specific examples of activities cited by the Costa Rican STEPS team include the adoption of national guidelines for care of patients with diabetes, hypertension, and dyslipidemia; establishment of a national screening program and regular risk factor surveillance; and provision of medications to healthcare centers. At the level of the health system, these factors (e.g., health insurance coverage, availability of diagnostic tests, and preventing drug stock-outs) may be important targets for countries looking to improve their health system performance for diabetes.

In addition to the individual-level predictors we explored, which included sex, age, educational attainment, household wealth quintile, and BMI, several other factors may influence how patients interact with the health system. These include, for example, lack of awareness of diabetes, low level of health-seeking behaviors, low risk perception for diabetes, and poor medication adherence. These factors are likely to be context-specific and dynamic, and should be explored in greater depth at the country level.

There are several limitations to this study. First, our sample includes data from 28 LMICs and thus patterns identified here may differ in countries that are not represented in this sample. Nonetheless, these 28 LMICs represent 48% of the world population and 57% of the population living in LMICs in 2016. Second, our definition of diabetes is based on only 1 glucose measurement. The lack of HbA1c (in all but 4 of the 28 countries) and an oral glucose tolerance test (OGTT) may have resulted in inaccuracy of the prevalence estimates for diabetes, and in particular the absence of OGTT may have resulted in an underestimate of diabetes prevalence [34]. Furthermore, diabetes was assessed on capillary blood in many countries. While modern glucometers generally electronically adjust capillary glucose values to plasma glucose values, the adjustment can be inaccurate, and definite diagnosis of diabetes should ideally be based on plasma glucose. The availability of only 1 glucose measurement also presented challenges to measuring control, though we ultimately chose a very liberal definition for controlled disease that should result in an overestimate of rates of control. Third, our data on respondent wealth were limited, which may have resulted in measurement error in this predictor and may partially explain the relatively weak association between cascade performance and wealth as compared to, for example, educational attainment. Fourth, while we included the most recent eligible survey for all countries, this was a 2009 survey for 5 countries (Chile, China, Fiji, Mongolia, and Mexico) and a survey about 10 years old for Benin (2008). Regular surveillance of these indicators is needed to provide up-to-date estimates to inform targeted interventions and policies.

There are several approaches to constructing the cascade of care, and we have used 1 approach [9]. Many studies in this area have relied on conditional probabilities, but we felt that a fixed denominator would provide a more policy-relevant analysis of where the loss to follow-up currently occurs for those with diabetes in LMICs. Additionally, our analysis of the “screening” stage in the cascade is limited by specific aspects of survey design. For example, most surveys included in the study asked participants about *ever* having received a glucose test. As such, we were unable to provide a more nuanced analysis of how recently or frequently respondents had been tested for diabetes. Moreover, the responses to this question were self-reported and thus subject to recall bias. One might expect those participants who were receiving treatment for diabetes were more likely to recall having been tested. It may also be the case that some participants who reported being tested for diabetes but not diagnosed did not actually meet the criteria for a diagnosis of diabetes at the time of the test but did so at the time of the survey. The low response rates for some of the surveys may have further impacted these self-report biases, and improving response rates for these national surveys should be a priority for future studies. These limitations should be considered when interpreting these findings.

Though not unique to this analysis, there are also limitations to the cascade of care approach as a tool for understanding health system performance. For instance, the cascade of care is one approach to quantifying the progress of individuals with a particular condition as they travel through the health system, but it does not provide exhaustive insight about the many factors that may conspire to cause loss to care. In particular, this approach is limited in its ability to assess structural inequalities both within and between countries that may explain the patterns in both care and control that were uncovered in this analysis. Furthermore, the cascade of care can provide useful insight about health system performance but is not a priority-setting exercise per se. The cascade of care is intended to provide an evidence base for important and complex discussions about resource allocation, including context-specific questions about the trade-offs between diagnostic and treatment activities and the use of finite resources for diabetes services, as compared to services for other important chronic diseases, including conditions such as HIV that have historically had greater visibility and more external funding. Both cost-effectiveness analyses and multi-criteria decision analysis are superior tools for priority-setting and decisions about resource allocation within health systems [35,36].

In conclusion, we are witnessing an important, rapid epidemiological transition in LMICs in which diabetes is a growing health concern. As this epidemic accelerates, there is a critical window in which health policymakers and healthcare providers can establish robust, high-quality, sustainable care for diabetes and other highly prevalent cardiometabolic conditions. This analysis offers to our knowledge the first multi-country assessment across several regions of the world of health system performance in managing diabetes and provides important policy-relevant insight to improve health system performance for this disease. These findings have important implications for efforts to achieve universal health coverage, defined as the goal that all people receive essential health services without being exposed to financial hardship [37]. Diabetes is a potential tracer for examining health systems, and this study suggests that many countries, in particular low-income and lower-middle-income countries, face challenges in achieving universal health coverage [38]. Future research should focus on interventions to improve performance in management of diabetes across the care cascade to improve the effectiveness of care provided and health outcomes.

## Supporting information

S1 Appendix Data search process. (DOCX)

S2 Appendix Search methods for LMICs that did not have an eligible WHO STEPS survey. (DOCX)

S3 Appendix Country categories and country-specific sampling methods. (DOCX)

S4 Appendix Detailed methodology for household wealth index calculation. (DOCX)

S5 Appendix Detailed methodology for diabetes biomarkers by country. (DOCX)

S6 Appendix Select questions from a generic STEPS instrument used in constructing the diabetes cascades of care. (DOCX)

S7 Appendix Percent (*n*) of participants with diabetes missing predictor variables, by country. (DOCX)

S8 Appendix Prevalence (95% confidence interval) of clinical diabetes by country. (DOCX)

S9 Appendix Sensitivity analyses of diabetes prevalence and cascade performance assuming all participants who were missing data on fasting status were non-fasting or excluding India. (DOCX)

S10 Appendix Cascade of care for diabetes (95% confidence interval) by country. (DOCX)

S11 Appendix Univariable regression analyses assessing the relationship between sociodemographic characteristics and testing, treatment, and control of diabetes in 28 LMICs. (DOCX)

S1 Checklist STROBE checklist. (DOCX)

S1 Text Country-specific contact information regarding accessing data used in this study. (DOCX)

## Abbreviations

BMI: body mass index
DHS: Demographic and Health Survey
LMICs: low- and middle-income countries
NCD: non-communicable disease
NFHS: National Family Health Survey
STEPS: Stepwise Approach to Surveillance
WHO: World Health Organization
