# Supplementary material for: Health system performance for people with diabetes in 28 low- and middle-income countries: A cross-sectional study of nationally representative surveys
Source: PLoS Med. 2019 Mar 1;16(3):e1002751. doi: 10.1371/journal.pmed.1002751 (PMC6396901; doi:10.1371/journal.pmed.1002751)
Supplement: S2 Appendix — (DOCX) [file pmed.1002751.s002.docx]

# Appendix 2: Search methods for low- and middle-income countries that did not have an eligible WHO STEPS survey

Search engine: Google

Search terms: “[country name]” AND (“population-based” OR household) AND (“blood glucose” OR “plasma glucose” OR “blood sugar” OR hemoglobin OR haemoglobin OR A1c OR HbA1c OR A1C OR Hb1c OR Hba1c OR HGBA1C OR “blood pressure” OR hypertension OR hypertensive OR cholesterol OR LDL OR HDL OR lipoprotein OR triglycerides OR triglyceride OR lipid OR lipids)

Number of hits reviewed: Hits reviewed until eligible survey identified, or, in the case of no eligible survey identified, first 500 hits (10 hits per page/5 pages reviewed)

Inclusion criteria for a survey:

1. The survey was conducted during or after 2005; in cases where two surveys were available for a particular country, the most recent was used;
2. The survey data were made available at the individual level;
3. The survey contained a biomarker for diabetes (either a glucose measurement or HbA1c);
4. The survey was conducted in an upper-middle, lower-middle or low-income country according to the World Bank at the time the survey was conducted;
5. The survey was nationally representative;
6. The survey included a suite of questions that assessed access to a core and comparable group of health services for diagnosis, preventive counselling, and treatment of diabetes.

Countries included in search: Afghanistan, Albania, Algeria, American Samoa, Angola, Argentina, Armenia, Azerbaijan, Bangladesh, Belarus, Belize, Bolivia, Bosnia and Herzegovina,

Brazil, Bulgaria, Burundi, Cameroon, Central African Republic, Chad Chile, China, Colombia, Congo, Cook Islands, Côte d'Ivoire, Cuba, Democratic People's Republic of Korea, Democratic Republic of the Congo, Djibouti, Dominica, Dominican Republic, Ecuador, Egypt, El Salvador, Equatorial Guinea, Eritrea, Fiji, Gabon, Ghana, Guatemala, Guinea, Guinea-Bissau, Haiti, Honduras, India, Indonesia, Jamaica, Kazakhstan, Kyrgyzstan, Lao People's Democratic Republic, Lesotho, Libya, Macedonia, Madagascar, Malaysia, Maldives, Mali, Marshall Islands, Mauritania, Mauritius, Mexico, Micronesia (Federated States of), Montenegro, Morocco, Namibia, Nicaragua, Niger, Nigeria, Occupied Palestinian Territory, Pakistan, Panama, Peru, Philippines, Romania, Russia, Saint Lucia, Samoa, São Tomé and Principe, Senegal, Serbia, Solomon Islands, Somalia, South Africa, South Sudan, Sudan, Suriname, Syrian Arab Republic, Tajikistan, Thailand, Tunisia, Turkey, Turkmenistan, Ukraine, Venezuela, Yemen, Zambia, Zimbabwe
