# Supplementary material for: Health system performance for people with diabetes in 28 low- and middle-income countries: A cross-sectional study of nationally representative surveys
Source: PLoS Med. 2019 Mar 1;16(3):e1002751. doi: 10.1371/journal.pmed.1002751 (PMC6396901; doi:10.1371/journal.pmed.1002751)
Supplement: S3 Appendix — (DOCX) [file pmed.1002751.s003.docx]

# Appendix 3: Country categories and country-specific sampling methods

**Country Categories**

| Low-Income | Benin, Burkina Faso, Comoros, Liberia, Nepal, Tanzania, Togo, Uganda |
| --- | --- |
| Lower-middle Income | Bangladesh, Bhutan, Georgia, India, Indonesia, Kenya, Mongolia, Swaziland, Timor-Leste |
| Upper-middle Income | Chile, China, Costa Rica, Fiji, Guyana, Mexico, Namibia, Romania, Seychelles, South Africa, St. Vincent & the Grenadines |
| Sub-Saharan Africa | Benin, Burkina Faso, Comoros, Kenya, Liberia, Namibia, Seychelles, South Africa, Swaziland, Tanzania, Togo, Uganda |
| Latin America & Caribbean | Chile, Costa Rica, Guyana, Mexico, St. Vincent & the Grenadines |
| South & Southeast Asia | Bangladesh, Bhutan, China, Fiji, India, Indonesia, Mongolia, Nepal, Timor-Leste |
| Middle East & Central Asia | Georgia, Romania |

**Country-Specific Sampling Methods**

*Note: In order to ensure accuracy in reporting, sampling methods are pasted verbatim from specified sources.*

**Bangladesh: DHS 2011**

“The sample for the 2011 BDHS is nationally representative and covers the entire population residing in noninstitutional dwelling units in the country. The survey used as a sampling frame the list of enumeration areas (EAs) prepared for the 2011 Population and Housing Census, provided by the Bangladesh Bureau of Statistics (BBS). The primary sampling unit (PSU) for the survey is an EA that was created to have an average of about 120 households. Bangladesh has seven administrative divisions: Barisal, Chittagong, Dhaka, Khulna, Rajshahi, Rangpur, and Sylhet. Each division is subdivided into zilas, and each zila into upazilas. Each urban area in an upazila is divided into wards, and into mohallas within a ward. A rural area in the upazila is divided into union parishads (UP) and mouzas within a UP. These divisions allow the country as a whole to be easily separated into rural and urban areas. The survey is based on a two-stage stratified sample of households. In the first stage, 600 EAs were selected with probability proportional to the EA size, with 207 clusters in urban areas and 393 in rural areas. A complete household listing operation was then carried out in all the selected EAs to provide a sampling frame for the second-stage selection of households. In the second stage of sampling, a systematic sample of 30 households on average was selected per EA to provide statistically reliable estimates of key demographic and health variables for the country as a whole, for urban and rural areas separately, and for each of the seven divisions. […] In one in three households selected in the 2011 BDHS survey, all ever-married men age 15-54 were selected and interviewed for the male survey. In this subsample, all woman and men age 35 and older were eligible to participate in the biomarker component, which included blood pressure measurements, testing for anemia, blood glucose testing, and height and weight measurements. […] Among these individuals, 92 percent of women and 86 percent of men participated in the blood pressure measurement, and 89 percent of women and 83 percent of men participated in the blood glucose measurement.”

*Source: National Institute of Population Research and Training (NIPORT), Mitra and Associates, and ICF International. 2013. Bangladesh Demographic and Health Survey 2011. Dhaka, Bangladesh and Calverton, Maryland, USA: NIPORT, Mitra and Associates, and ICF International.*

**Benin: STEPS 2008**

“The STEPS survey in Benin was a population-based survey of adults aged 25-64. A cluster sample design was used to produce representative data for that age range. A total of 6,904 adults participated in the Benin STEPS survey. Recruitment was based on a random five-stage sampling frame. Sixty of 546 districts were randomly selected according to the sizes of their populations. In each district retained, a list of neighborhoods or villages was drawn up and half were selected. In each neighborhood retained, dwellings, households, and then subjects were randomly selected. An investigator went to the center of each neighborhood or village and randomly chose a direction to go before entering one out of every two dwellings. In the dwellings retained, he listed the households and randomly selected one out of two. Within each household, the participant was identified using the Kish method. This procedure was followed until the predetermined sample was obtained for the neighborhood or village concerned. The response rate for the survey was 99%. With respect to the biological data collected in STEP 3, this module was] systematically proposed to six subjects out of ten."

*Source: Houehanou YC, Lacroix P, Mizehoun GC, Preux PM, Marin B, Houinato DS. Magnitude of cardiovascular risk factors in rural and urban areas in Benin: findings from a nationwide steps survey. PLoS One 2015; 10(5): e0126441.*

**Bhutan: STEPS 2014**

“To achieve a nationally representative sample, a multistage sampling method was used to select enumeration areas, households and eligible participants at each of the selected households in three stages. The 2005 National Census was chosen as the basis for the sampling frame, with “Geogs” (blocks) in rural areas and towns in urban areas forming the primary sampling units (PSUs). Since the population distribution for urbanicity is 70:30 (rural:urban), 63 PSUs in rural and 14 PSUs in urban areas were chosen. PSUs were selected through the probability proportionate to size (PPS) sampling using the number of households in each PSU. Two secondary sampling units (SSUs) for every rural PSU and 4 SSUs for every urban PSU were selected. This led to the selection of 126 SSUs from rural and 56 SSUs from urban areas. This was also carried out by PPS sampling, using the number of households in each SSU. A total of 16 households from each SSU (both rural and urban) were selected using systematic random sampling. The sampling frame for this was the list of households with a unique identification number (ID) developed by the enumerators for the survey. At the household level, the Kish sampling method was used to randomly select one eligible member (aged 18–69 years) of the household for the survey. The Kish method ranks eligible household members in order of decreasing age, starting with males and then females, and randomly selects a respondent using the automated program for Kish selection in the handheld personal digital assistant (PDA).”

*Source: National survey for noncommunicable disease risk factors and mental health using approach WHO Steps Approach in Bhutan – 2014 Available at: http://www.who.int/chp/steps/bhutan/en/.*

**Burkina Faso: STEPS 2013**

“Sampling methodology: The study was conducted on a sample obtained from a three-stage cluster stratified as recommended by the WHO for STEPS screening surveys. risk factors for noncommunicable diseases.

The sampling frame used was that derived from the general census of the population and habitat 2006 (RGPH 2006) and updated in 2010 during the survey Demographic and Health Survey of Burkina Faso (EDS-BF, 2010). This update concerned the enumeration areas (EAs) that correspond to the cluster as part of this study.

Selection of clusters: The choice of clusters was made according to a systematic random selection proportional to their size (in number of households) within strata (regions). To do this clusters were organized by stratum and place of residence (urban / rural). A total of 240 clusters of which 185 were in rural areas and 55 in urban areas were selected for the investigation.

Selection of households: Households were randomly drawn after an enumeration exhaustive list of all households in the cluster. A draw tool designed on Excel by the team. The technique was used in the field for selecting households to investigate. In total, 20 households in clusters were selected to participate in the study.

Selection of individuals: The choice of individuals was made randomly using Kish's method. In total, an individual aged 25 to 64 living in a selected household was fired for participate in the survey.”
*Source, translated from: Rapport de l’enquete national sur la prevalence des principaux facteurs de risques communs aux maladies non transmissibles au Burkina Faso Enquete STEPS 2013. Available at: http://www.who.int/chp/steps/burkina_faso/en/.*

**Chile: NHS 2009-10**

“The sampling frame was constituted from the Population and Housing Census 2002. The design of the study was transversal, with a random sample of complex type households (stratified and multi-stage by clusters) with national, regional and area representation rural / urban. The target population was adults older than or equal to 15 years. The survey had a response rate in the eligible population of 85%. The refusal rate was of 12%. 5,434 people were interviewed. A nurse performed clinical and examinations to 5,043 participants and 4,956 accepted laboratory tests (blood and urine). The total sample loss of the oversized sample was 28% (this including rejection, non-contact and other causes of random loss). The raw sample was designed with overrepresentation of some population groups (older adults, regions other than the Metropolitan Region and rural areas) to increase sample efficiency and homogenize the accuracy of the estimators. The expansion of the sample data is because it grants each participant the weight that corresponds to it according to the design sample and at the same time corrects the distortion of the raw sample, making it coincide with the census population projection for January 2010 for Chilean adults over 15 years of age.“

*Source, translated from: Resumen Ejecutivo: Encuesta Nacional de Salud ENS Chile 2009-10. Available at: http://epi.minsal.cl/encuesta-ens-anteriores/.*

**China: CHNS 2009**

“The China Health and Nutrition Survey is a longitudinal study across 228 communities within nine provinces of China. Surveys began in 1989, with subsequent surveys every 2–4 years, for a total of nine rounds between 1989 and 2011. The China Health and Nutrition Survey was designed to provide representation of rural, urban and suburban areas varying substantially in geography, economic development, public resources and health indicators,[^13^](https://www.ncbi.nlm.nih.gov/pmc/articles/PMC4491857/#bib13) and it is the only large-scale, longitudinal study of its kind in China. The original survey in 1989 used a multistage, random cluster design in eight provinces (Liaoning, Jiangsu, Shandong, Henan, Hubei, Hunan, Guangxi and Guizhou) to select a stratified probability sample; a ninth province, Heilongjiang, was added in 1997 using a similar sampling strategy. Essentially, two cities (one large and one small city—usually the provincial capital and a lower income city) and four counties (stratified by income: one high, one low and two middle income counties) were selected in each province. Within cities, two urban and two suburban communities were selected; within counties, one community in the capital city and three rural villages were chosen. Twenty households per community were then selected for participation. The study met the standards for the ethical treatment of participants and was approved by the Institutional Review Boards of the University of North Carolina at Chapel Hill and the Institute of Nutrition and Food Safety, Chinese Center for Disease Control and Prevention.”

*Source: Attard, Samantha M.; Herring, Amy H.; Wang, Huiling; Howard, Annie Green; Thompson, Amanda L.; Adair, Linda S.; Mayer-Davis, Elizabeth J.; & Gordon-Larsen, Penny. (2015). Implications of Iron Deficiency/Anemia on the Classification of Diabetes Using HbA1c. Nutrition & Diabetes, 5, e166.*

**Comoros: STEPS 2011**

“The STEPS survey on risk factors for chronic diseases in the Union of the Comoros took place from January to March 2011. This study has undertaken Step 1, Step 2 and Step 3. Indeed, socio-demographic and behavioral measures were collected in Step 1. Physical measures such as height, weight and tension were collected in Step 2 and biochemical measurements were collected to assess the levels of blood glucose and cholesterol levels in Step 3. The STEPS survey conducted in Comoros Union is a survey of general population, targeting adults aged 25 to 64 years. A stratified survey was used to produce representative data for this age group. A total of 5556 adults aged 25 to 64 participated in the STEPS survey on a sample of 5760 people representing an overall response rate of 96.5%.”

*Source, translated from* Union des Comores STEPS 2011 Note de synthèse.

*Available at: http://www.who.int/chp/steps/comoros/en/.*

**Costa Rica: STEPS 2010**

“The Costa Rican NCRFSS survey was a cross-sectional survey based on a probabilistic cluster sampling design. The NCRFSS survey was conducted during 2010 under the supervision of the Caja Costarricense de Seguro Social, a government public healthcare provider, and covers the overall adult population aged ≥20 years. Multistage cluster sampling was performed stratified by geographical areas, age groups (20–39, 40–64, and ≥65 years) and gender. The first sample stage was the randomized selection of the country’s geographical areas as primary sample units followed by the random selection of sectors in selected areas as secondary sample units. The random selection of areas and sectors was performed with probability proportional to size; the area or sector size was determined by the population >20 years during 2009, as estimated by the Costa Rican Census and Statistics National Institute (INEC). Households were chosen through a random number generator using dwelling lists obtained from the health technician assistant in every community until all age group and gender strata sample sizes were achieved. A family dwelling was defined as a group of people who share the same table to eat. Survey participants were selected by the Kish method, which samples participants within a household with equal probability of selection, as recommended by the WHO STEPwise methodology. To be eligible for inclusion in the study, subjects had to be ≥20 years of age, permanently residing in the selected homes, and to have provided written consent. Pregnant or lactating mothers and those who were within 6 months postpartum were excluded from the study. Each participant selected for the study was informed of the study objectives and details before agreeing to participate in the investigation. In all, 3653 noninstitutionalized adults were surveyed, with an 87.8% response rate of the eligible population.”

*Source: Wong-McClure R, Gregg EW, Barcelo A, Sanabria-Lopez L, Lee K, Abarca-Gomez L, Cervantes-Loaiza M, Luman ET. Prevalence of diabetes and impaired fasting glucose in Costa Rica: Costa Rican National Cardiovascular Risk Factors Survey, 2010. J Diabetes. 2016 Sep;8(5):686-92.*

**Fiji: EHS 2009**

“The sample frame (188 800 people aged ≥40 years; 50.3% female; 49.4% Melanesian Fijian,

44.9% Indo-Fijian, and 5.7% of other ethnicity; 43.2% rural dwellers) included all 8 provinces of Viti Levu, Fiji’s main island, where 79.1% of the total population resides. Using an anticipated prevalence of vision impairment of 11.0% in the target population (actual was 11.4%; 95% confidence interval [CI] = 9.9% to 13.2%), absolute precision of ±2.2% (20% relative difference), with 95% confidence, a design effect of 1.4 and a response rate of 80%, the sample size was determined to be 1354 persons. From the sample frame, 34 clusters of 40 people were required. Across Viti Levu, the clusters were selected through probability proportionate to size sampling, using national census data.”

*Source: pasted verbatim from email exchange with study team.*

**Georgia: STEPS 2016**

“The STEPS survey of noncommunicable disease (NCD) risk factors in Georgia was carried out from June 2016 to September 2016. Georgia carried out Step 1, Step 2 and Step 3. Socio demographic and behavioural information was collected in Step 1. Physical measurements such as height, weight and blood pressure were collected in Step 2. Biochemical measurements were collected to assess blood glucose and cholesterol levels in Step 3. The survey was a population-based survey of adults aged 18-69. A Multi-stage cluster sampling design was used to produce representative data for that age range in Georgia. A total of 5554 adults participated in the survey. The overall response rate was 75.7%.”

*Source: Georgia STEPS Survey 2016 Fact Sheet.*

*Available at: http://www.who.int/chp/steps/georgia/en/.*

**Guyana: STEPS 2016**

“A response rate of 66.68% will be selected based on the experience and response rates of other surveys over the years such as the recent Demographic Health Survey 2009. [...] STEPS 3 involve taking blood samples from a proportion of the sample, in this case 50% of the sample, in order to measure raised blood glucose levels and abnormal blood lipids. [...] The STEPS sample will be prepared by the Bureau of Statistics Guyana following the recommended STEPS sample methodology. A multi-stage cluster sampling design will be used. Guyana is divided into 10 administrative regions and within the administrative regions there are seven towns and each region is further divided into enumeration districts. For the STEPS survey 288 enumeration districts will be selected using the population probability sampling method and from each enumeration district 12 households will be selected giving a total sample size of 3456. Further at the household level each participant will be randomly selected by the electronic tablet. For STEP 3 50% of the sample will be randomly selected to participate. A re-listing of some households may also be necessary, such as those interior region locations, in which case in addition to household listings, enumeration districts maps will also be provided so that a re-listing can be done where required.”

*Source: STEPwise Approach to Chronic Disease risk factor surveillance (STEPS): Guyana’s Implementation Plan. June 20, 2016. Ministry of Public Health, Guyana.*

**India: NFHS 2015-16**

“The NFHS-4 sample was designed to provide estimates of all key indicators at the national and state levels, as well as estimates for most key indicators at the district level (for all 640 districts in India, as of the 2011 Census). The total sample size of approximately 572,000 households for India was based on the size needed to produce reliable indicator estimates for each district and for urban and rural areas in districts in which the urban population accounted for 30-70 percent of the total district population. The rural sample was selected through a two-stage sample design with villages as the Primary Sampling Units (PSUs) at the first stage (selected with probability proportional to size), followed by a random selection of 22 households in each PSU at the second stage. In urban areas, there was also a two-stage sample design with Census Enumeration Blocks (CEB) selected at the first stage and a random selection of 22 households in each CEB at the second stage. At the second stage in both urban and rural areas, households were selected after conducting a complete mapping and household listing operation in the selected first-stage units.”

*Source:* *Ministry of Health and Family Welfare (MoHFW) - Government of India.* *India - National Family Health Survey 2015-2016. Report generated on: February 7, 2018.*

**Indonesia: IFLS 2014-15**

“Because it is a longitudinal survey, IFLS5 drew its sample from IFLS1, IFLS2, IFLS2+, IFLS3 and IFLS4. The IFLS1 sampling scheme stratified on provinces and urban/rural location, then randomly sampled within these strata (see Frankenberg and Karoly, 1995, for a detailed description). Provinces were selected to maximize representation of the population, capture the cultural and socioeconomic diversity of Indonesia, and be costeffective to survey given the size and terrain of the country. For mainly cost-effectiveness reasons, 14 of the then existing 27 provinces were excluded.3 The resulting sample included 13 of Indonesia’s 27 provinces containing 83% of the population: four provinces on Sumatra (North Sumatra, West Sumatra, South Sumatra, and Lampung), all five of the Javanese provinces (DKI Jakarta, West Java, Central Java, DI Yogyakarta, and East Java), and four provinces covering the remaining major island groups (Bali, West Nusa Tenggara, South Kalimantan, and South Sulawesi).

Within each of the 13 provinces, enumeration areas (EAs) were randomly chosen from a nationally representative sample frame used in the 1993 SUSENAS, a socioeconomic survey of about 60,000 households. The IFLS randomly selected 321 enumeration areas in the 13 provinces, over-sampling urban EAs and EAs in smaller provinces to facilitate urban-rural and Javanese–non-Javanese comparisons.

Within a selected EA, households were randomly selected based upon 1993 SUSENAS listings obtained from regional BPS office. A household was defined as a group of people whose members reside in the same dwelling and share food from the same cooking pot (the standard BPS definition). Twenty households were selected from each urban EA, and 30 households were selected from each rural EA. This strategy minimized expensive travel between rural EAs while balancing the costs of correlations among households. For IFLS1 a total of 7,730 households were sampled to obtain a final sample size goal of 7,000 completed households. This strategy was based on BPS experience of about 90% completion rates. In fact, IFLS1 exceeded that target and interviews were conducted with 7,224 households in late 1993 and early 1994.

In IFLS1 it was determined to be too costly to interview all household members, so a sampling scheme was used to randomly select several members within a household to provide detailed individual information.”

*Source: Strauss, J., F. Witoelar, and B. Sikoki. “The Fifth Wave of the Indonesia Family Life Survey (IFLS5): Overview and Field Report”. March 2016. WR-1143/1-NIA/NICHD.*

**Kenya: STEPS 2015**

“The 2015 Kenya STEPs survey was a national cross-sectional household survey designed to provide estimates for indicators on risk factors for non-communicable diseases for persons age 18 – 69 years. The sample was designed with a sample size of 6,000 individuals to allow national estimates by sex (male and female) and residence (urban and rural areas). The survey used the fifth National Sample Surveys and Evaluation Programme (NASSEP V) master sample frame that was developed and maintained by KNBS. The frame was developed using the Enumeration Areas (EAs) generated from the 2009 Kenya Population and Housing Census to form 5,360 clusters split into four equal sub-samples. A three-stage cluster sample design was adopted for the survey involving selection of clusters, households and eligible individuals. In the first stage, 200 clusters (100 urban and 100 rural) were selected from one sub-sample of NASSEP V frame. A uniform sample of 30 households from the listed households in each cluster was selected in the second stage of sampling. The last stage of sampling was done using Personal Digital Assistants (PDAs) at the time of survey, where one individual was randomly selected from all eligible listed household members using a programmed KISH method of sampling.”

*Source: WHO: Kenya STEPwise Survey for Non Communicable Diseases Risk Factors 2015 Report. Available at: http://www.who.int/chp/steps/Kenya_2015_STEPS_Report.pdf?ua=1.*

**Liberia: STEPS 2011**

“Random multi-cluster sampling method was used to collect data during this survey in 5 of the 15 counties of Liberia with the district serving as the primary sampling unit. Different sampling frames were designed and used at the district (Primary Sampling Unit-PSU), Chiefdoms (Secondary Sampling Unit-SSU) and household levels. Households listing generated from the 2008 National Population Census was used, and in each household, the list of individuals’ resident was obtained and the Kish Method was used. Kish Method is a household sampling technique developed by WHO for STEPS. The field team selected households by using nutrition sampling method (throwing a pencil to get a selected direction). When the household enumeration sampling point is established, the interviewer counts all the households and using interval sample to get the household number. In each household, one person was selected using the Kish method.”

*Source: WHO: The Final Report on the Liberia STEPS Survey 2011. Available at: http://www.who.int/chp/steps/Liberia_2011_STEPS_Report.pdf?ua=1.*

**Mexico: MxFLS 2009-12**

“The design of the first round, the baseline survey (MxFLS-1), was undertaken by the National Institute of Statistics and Geography (INEGI, per its name in Spanish).  The baseline sample is probabilistic, stratified, multi-staged, and independent at every phase of the study.  The population is comprised by Mexican households in 2002. Primary sampling units were selected under criterions of national, urban-rural and regional representation on pre-established demographic and economic variables. Regional definitions are in accordance with the National Development Plan 2000-2006.

Currently, the MxFLS contains information for a 10-year period, collected in three rounds: 2002 (MxFLS-1), 2005-2006 (MxFLS-2) and 2009-2012 (MxFLS-3). Future rounds have been programmed in order to have a database that allows studying efficiently the well-being of the Mexican population at different moments in time. The first round or baseline survey (MXFLS-1), implemented in 2002, collected information on a sample of 35,000 individuals from 8,400 households in 150 communities throughout the country. The second (MxFLS-2) and third round (MxFLS-3) were conducted during 2005-2006 and 2009-2012, respectively. Given the longitudinal design of the survey, the MxFLS-2 and MxFLS-3 aimed to relocate and reinterview the sample of the MxFLS-1—including those individuals who migrated within Mexico or emigrated to the United States of America—and to interview the individuals or households that grew out from previous samples. The MxFLS-2 and MxFLS-3 relocated and reinterviewed almost 90 percent of the original sampled households. A primary goal of the Mexican Family Life Survey (MxFLS) is to create a longitudinal and multi-thematic database. On the one hand, the longitudinal design allows a long term tracking of individuals regardless of changes in residence and new household formations (split-offs). On the other hand, the multi-thematic design allows collecting—with a single tool—a wide range of socioeconomic and demographic indicators of the Mexican population. The first round of the survey (MxFLS-1) took place during 2002 reaching a sample of 8,400 households (35,000 individuals) in 150 urban and rural communities throughout the country. The second (MxFLS-2) and third round (MxFLS-3) were conducted during 2005-2006 and 2009-2012, respectively. Given the longitudinal design of the survey, the MxFLS-2 and MxFLS-3 aimed to relocate and reinterview the sample of the MxFLS- 1—including those individuals who migrated within Mexico or emigrated to the Unites States—and to interview the individuals or households that grew out from previous samples. The MxFLS-2 and MxFLS-3 relocated and reinterviewed almost 90 percent of the original sampled households.”

*Source: The Mexican Family Life Survey website. http://www.ennvih-mxfls.org/english/introduccion.html. Accessed 16 November 2017.*

**Mongolia: STEPS 2009**

“A total of 5,438 randomly selected 15-64 year-old Mongolian residents of both sexes from 36 soums of 20 aimags and 24 khoroos of 6 districts of Ulaanbaatar city participated in the survey. All participants completed STEPS 1 and 2 of the survey except pregnant women, persons with disabilities and bedridden patients, for whom physiological measurements were not performed.

Every third person aged 25-64 randomly selected into the study completed STEP 3 or biochemical testing. Response rate of the survey was 95% in both STEP1, 2 and STEP3.

The survey was designed to cover all geographical areas of Mongolia, and a four-stage cluster sampling process was carried out to randomly select participants from the target population. Given the urban vs. rural differences in lifestyle and disease status, the target population was stratified into urban and rural areas and the sample was drawn proportionally from each based on the target population in each area. Thus, out of the 60 total clusters selected, 28 were urban and 32 were rural. Ulaanbaatar city, Darkhan-Uul and Orkhon aimags represented urban areas, while the rest of aimags and soums represented rural areas (Table 1).

**Table 1. Number of urban and rural clusters**

| Setting | Target population | As % of total population | Survey population | Number of clusters |
| --- | --- | --- | --- | --- |
| Urban | 831802 | 0.48 | 2733 | 28 |
| Rural | 919417 | 0.52 | 2961 | 32 |
| Total | 1751219 | 1.00 | 5694 | 60 |

Ninety-five participants were selected from each cluster. In the urban areas, khoroos were selected, then family groups, then households. In rural areas, soums were selected, then baghs then households How this selection was proceeded is explained in the following paragraph.

A total of 28 khoroos were selected from a list of all khoroos (n=173) in the urban area using probability proportional to size sampling, where the size was 25.5% of the total target population of 851,062 persons aged 15-64 years old. From each selected khoroo, three of the family groups were selected using probability proportional to its total number of households. Within each of the three family groups, 95 households were selected using simple random sampling from updated household registries.

For the rural area, 32 of the soums were selected from a list of all soums (n=324) using probability proportional to size sampling, where the size was 23.1% of the total target population of 919,417 persons aged 15-64 years old. From each selected soum, 2 baghs were selected using probability proportional to its total number of households. Within each selected baghs, 95 of the households were selected using simple random sampling from updated household registries.

From each selected household in urban and rural areas, only one person aged 15-64 years was selected using Kish Method as the last step.”

*Source: MONGOLIAN STEPS SURVEY ON THE PREVALENCE OF NONCOMMUNICABLE DISEASE AND INJURY RISK FACTORS – 2009. Available from: http://www.who.int/ncds/surveillance/steps/2009_STEPS_Report_Mongolia.pdf*

**Namibia: DHS 2013**

“The sample for the 2013 NDHS was a stratified sample selected in two stages. In the first stage, 554 EAs were selected with a stratified probability proportional to size within the sampling frame. The EA size is the number of households residing in the EA and recorded in the 2011 NPHC. Stratification was achieved by separating each region into urban and rural areas. Therefore, the 13 regions were stratified into 26 sampling strata: 13 rural strata, and 13 urban strata. Samples were selected independently in each stratum, with a predetermined number of EAs selected as shown in Table A.3. Implicit stratification with proportional allocation was achieved at each of the lower administrative unit levels by sorting the sampling frame before the sample selection. Sorting was done according to the constituency and the EA code within a sampling stratum, and by using a probability proportional-to-size selection procedure.

After the selection of EAs and before the main survey, a household listing operation was carried out in all selected EAs, and the resulting lists of households served as a sampling frame for the selection of households in the second stage. Some of the selected EAs may large. To limit the amount of work done to list each household, selected EAs with more than 200 households were segmented by the listing team in the field before the household listing. Only one segment was selected for the survey, with probability proportional to the segment size. Household listing was conducted only in the selected segment (see detailed instructions for segmentation in the DHS Manual for Household Listing). So a 2013 NDHS cluster is either an EA or a segment of an EA. In the second-stage selection, a fixed number of 20 households was selected in every urban cluster and rural cluster, by equal probability systematic sampling. A spreadsheet indicating the selected household numbers for each cluster was prepared. The survey interviewers interviewed only the pre-selected households. To prevent bias, no replacements and no changes of the pre-selected households were allowed in the implementing stages. In half of the selected households where there was no male survey, all women age 15-49 were interviewed; in the other half of the selected households where there was a male survey, all males and females age 15-64 were interviewed.”

*Source: The Nambia Ministry of Health and Social Services (MoHSS) and ICF International. 2014. The Namibia Demographic and Health Survey 2013. Windhoek, Namibia, and Rockville, Maryland, USA: MoHSS and ICF International.*

**Nepal: STEPS 2013**

“The surveyed population included men and women aged 15–69 years who had been living at their place of residence for at least six months. [...]

The sample size was calculated to represent the entre target population in Nepal. In order to achieve this statistical inference, the sample size calculator by WHO (sample_size_calculator STEPS) was used to derive a sample size of 4,200. […]

Probability proportionate to size (PPS) was applied in the sampling strategy to improve the precision of the survey estimates. [...]

For this survey, the Ilaka was taken as the primary sampling unit (PSU). Out of the 921 Ilakas in Nepal, 159 are in the mountains, 467 in the hills and 295 in the Terai. The Steering Committee and the WHO NCD STEPS team at WHO headquarters in Geneva predetermined the number of PSUs to be taken in the study as 70. Thus, 70 Ilakas were sampled. Considering the varied distribution of the population across the ecological belts and to avoid the risk of under selection of the sample from the sparsely populated mountain belt, the distribution of ""Ilakas across ecological belts was determined on the basis of the population distribution pattern in the ecological belts (mountains 7%, hills 43% and Terai 50%). Hence, 30 Ilakas were selected from the hills, 5 from the mountains and 35 from the Terai using PPS. [...]

For the survey, wards (sub-units of VDCs and municipalities) were considered as clusters and taken as the secondary sampling unit (SSU). Three clusters were selected from each of the sampled Ilakas using PPS. All wards for each of the selected Ilakas were listed in order according to their numeric code, then 210 wards were selected (3 wards from each of the 70 Ilakas). To select the three wards from the list, all of the wards in the Ilaka were given a unique identification number, listed in ascending order along with household size and populated in the software. The software then selected the wards randomly on the basis of PPS.

Twenty households were selected from each cluster using systematic sampling. Thus, a total of 4,200 households were selected from the 210 clusters (20 households per cluster or ward). The sampling interval was determined by dividing the total number of households in the selected wards by 20. [...]

In municipalities, one ward covers a large number of households and each ward has more than 5 and sometimes up to 100 streets (margs or toles). Two margs or toles were selected and ten households were selected from each of the two margs or toles using systematic random sampling. If two or more families were found living in a house, one family was selected randomly. Eligible candidates (15–69 years) from the selected household were listed according to age and sex (males first and then females, in descending order), which was then fed into the Kish program in the personal digital assistants (PDAs), which automatically randomly selected one eligible candidate from each house.”

*Source: WHO: Non Communicable Diseases Risk Factors: STEPS Survey Nepal 2013. Available at: http://www.who.int/chp/steps/nepal/en/.*

**Romania: SEPHAR II**

“Sampling was performed by a multi-stratified procedure, leading to the selection of a representative sample of 1942 adults. Subject selection followed the principle of equality of chances of being enrolled in the study, regardless of the size of the place of residency.

Stratification criteria for sample selection were:

- territorial regions (Romania's territory was divided into 7 regions plus the capital city Bucharest, based on the National Statistics Institute recommendations: the North-East region, the South-East region, the South region, the South-West region, the West region, the North-West region, the Central region and the Bucharest region);
- locality type (cities with over 200 000 inhabitants, cities with 50 000–200 000 inhabitants, cities with less than 50 000 inhabitants, Commune);
- gender (male and female);
- age groups (18–24 years, 25–34 years, 35–44 years, 45–54 years, 55–64 years, 65–80 years).

In the first stage of selection, the adult population weighted average was calculated for each region and each district, and, based on this, the number of adult persons from each region/district was calculated from the working sample of 1942 subjects.

In the second stage of selection, the number of localities of a certain size from which the subjects were later selected was established for each district. This number was directly proportional to the population in the respective district. A random selection of a certain locality in a certain category was done using a computer software (generation of random numbers). The selected localities represent the interview centers where the study was to take place. The weighted average of the specific locality population in the district was calculated, and, based on this, the number of people selected to participate in the study.

The third stage of selection consisted of distribution by gender of adult people selected from each locality, using Romania's population gender distribution according to the 2002 census (F : M = 51.25% vs. 48.75%) and the fourth stage of selection consisted of distribution by age of male and female adult people selected from each locality, using Romania's population age distribution according to the 2002 census.”

*Source: Dorobantu M, Tautu OF, Darabont R, Ghiorghe S, Badila E, Dana M, Dobreanu M, Baila I, Rutkowski M, Zdrojewski T. Objectives and methodology of Romanian SEPHAR II Survey. Project for comparing the prevalence and control of cardiovascular risk factors in two East-European countries: Romania and Poland. Arch Med Sci. 2015 Aug 12;11(4):715-23.*

**Seychelles: STEPS 2013**

“The survey was performed in a sex and age stratified random sample of all adults aged 25‐64 years of Seychelles between October and December 2013 on Mahé and during 2 weeks in February 2014 in the islands of Praslin and La Digue. These three islands account for >98% of the total population of Seychelles. The eligible sample was extracted from the population registry. The survey was attended by 1240 adults, with a participation rate of 73%. Participants were invited to attend the survey on selected days in study centers located in Mahé, Praslin, and La Digue. All the eligible participants who did not attend were actively traced using (telephone, local administration, announcements on radio, etc) and invited to attend the survey. Since participants were randomly selected from the general adult population, findings of the survey can be inferred to the general adult population of Seychelles.”

*Source: National Survey of Noncommunicable Diseases in Seychelles 2013‐2014 (Seychelles Heart Study IV): methods and main findings. Available at: http://www.who.int/chp/steps/seychelles/en/.*

**South Africa: SANHANES 2012**

“The survey applied a multi-stage disproportionate, stratified cluster sampling approach. A total of 1000 census enumeration areas (EAs) from the 2001 population census were selected from a database of 86,000 EAs and mapped in 2007 using aerial photography to create the 2007 HSRC master sample to use as a basis for sampling of households. The selection of EAs was stratified by province and locality type. In the formal urban areas, race was also used as a third stratification variable (based on the predominant race group in the selected EA at the time of the 2001 census). The allocation of EAs to different stratification categories was disproportionate, in other words, over-sampling or over-allocation of EAs occurred in areas that were dominated by Indian, coloured or white race groups to ensure that the minimum required sample size in those smaller race groups were obtained. Based on the HSRC 2007 Master Sample, 500 Enumerator Areas (EAs) representative of the sociodemographic profile of South Africa were identified and a random sample of 20 visiting points (VPs) were randomly selected from each EA, yielding an overall sample of 10 000 VPs. EAs were sampled with probability proportional to the size of the EA using the 2001 census estimate of the number of VPs in the EA database as a measure of size (MOS). One of the tasks of SANHANES-1 was to recruit and establish a cohort of 5 000 households to be followed up over the coming years. The sampling consisted of: Multi-stage disproportionate, stratified cluster sampling approach; 500 EAs within which 20 VPs/households per EA were sampled; Main reporting domains: sex (male, female), age-group (< 2 years, 2–5 years, 6–14 years, 15–24 years, 25–49 years, 50 years and older), race group (black African, white, coloured, Indian), locality type (urban formal, urban informal, rural formal [including commercial farms] and rural informal], and province (Western Cape, Eastern Cape, Northern Cape, Free State, KwaZulu-Natal, North West, Gauteng, Mpumalanga, Limpopo).”

*Source: Human Sciences Research Council. SANHANES: Health and Nutrition. 2015. Available at: http://www.hsrc.ac.za/en/research-areas/Research_Areas_PHHSI/sanhanes-health-and-nutrition*

**St. Vincent & the Grenadines: STEPS 2013**

“The survey covered the entire island St. Vincent and the Grenadines, and was conducted using the following zoning categories:

1) Mainland (St. Vincent)

2) Northern Grenadines (Bequia and Mustique)

3) Southern Grenadines (Canouan and Union Island)

The sample size was proportionately divided between the three main reporting strata (St.Vincent/Northern Grenadines/Southern Grenadines). The country’s most recent age breakdown based on the 2001 national census by St. Vincent was used to approximate the adult population 18-69 years by Island grouping. The survey was stratified by sex, age groups 18-29, 30-44 and 45-69 years and by geographical location – St. Vincent, Northern Grenadines and Southern Grenadines.

A three-stage cluster sampling approach was used. Enumeration districts were randomly selected using Probability Proportional to Size (PPS) from the sampling frame. A total of 199 enumeration districts were selected. The sampling frame was developed using the number of households per enumeration district taken from the 2012 preliminary census report; enumeration districts had been subsequently revised (2010-2011) so that no enumeration district containing more than 150 Households would be randomly selected from the selected enumeration districts. The number of households per enumeration district to be selected was 26. Where an enumeration district had been split into 2 or more new enumeration districts the number of households in the previously defined enumeration district was divided equally between the newly revised enumeration districts. The household list for each selected enumeration district was updated prior to selection of households during a re-listing exercise. This was necessary as the existing household listing for each enumeration district was outdated.

Eligible persons at the household level were randomly selected using the Kish method. If no one was present in the selected household, a notification of visit card was left and the interviewer revisited. There was a total of three visits to the household before it was listed as non-response (one initial recruitment visit and two call backs). The interviewer then moved on to the next house on the list in the original order. Although the person selected for interview were to be at least 18 years and not older than 69 years on the last birthday, there were a few instances where some participants were turning 18 or 70 years; those cases were addressed during data cleaning.

Biological samples, testing and Nutrition intake (24 hour recall):

Fifty percent (50%) of the survey participants were asked to provide a biological specimen (finger prick) for Glucose and cholesterol testing using Glucose and Lipid Sampling Kits and respond to the nutrition intake (24 hour recall). The biological sample was only collected with participants’ explicit consent; the samples were not stored or used for additional undetermined or undisclosed future testing to which respondents did not agree at the time of participation.”

*Source: WHO STEPS: Noncommunicable Disease Risk Factor Surveillance. Report for St. Vincent & the Grenadines 2015. Available at: http://www.who.int/ncds/surveillance/steps/stvincent/en/*

**Swaziland: STEPS 2014**

“A Multi-stage cluster sampling design was applied. The survey covered all the four regions of the country. The size of the country and the distances between the regions and communities made it possible for the survey to sample a population representing all the 4 regions. The Multi-stage sampling procedure was implemented in the following procedural steps:

Stage 1: All four regions were included as a sampling frame of our Primary Sampling Unit (PSU).The number of the PSUs at this stage ensured precision in the survey estimates and as a result 216 PSUs were selected using probability proportional to size sampling.

Stage 2: The second stage of cluster sampling procedure entailed listing, sorting and random systematic sampling of the Secondary Sampling Units (Households) within the PSUs selected in stage1 where 20 households were selected from each PSU. Based on census data, only households with eligible participants were systematically sampled through random systematic sampling.

Stage 3: At this level, all the eligible participants within a household were sequentially listed into the PDAs and only one participant per household was randomly sampled using KISH method built into the PDAs. The KISH method is a widely used technique that uses a pre-assigned table of random numbers to identify the person to be interviewed.”

*Source: WHO STEPS: Noncommunicable Disease Risk Factor Surveillance Report Swaziland 2014. Available at: http://www.who.int/chp/steps/swaziland/en/.*

**Tanzania: STEPS 2012**

"The STEPS survey in the United Republic of Tanzania was a population-based survey of adults aged 25-64. The study used both multistage cluster and random probability sampling procedures. Fifty of 119 total districts were randomly selected as primary sampling units (PSUs). Within these PSUs, enumeration areas (EAs) of > 50 households were randomly selected. Any EA with < 50 households was merged with a neighboring EA. Within the EAs, households were randomly selected from a list of all eligible households in the EA. A total of 5762 adults participated in the Tanzania STEPS survey. Within each selected household, the Kish method was used to select the STEPS participant. This procedure was followed until the predetermined sample was obtained for the enumeration area. The response rate for this survey was 94.7%.”

*Source: Tanzania STEPS Survey Report. Available at: http://www.who.int/chp/steps/UR_Tanzania_2012_STEPS_Report.pdf?ua=1*

**Timor-Leste: STEPS 2014**

“Note: Data from Census 2010 were used for all sampling considerations. Even though planning and mapping for 2015 Census is ongoing, data from the Census will only be available after July 2015.

STEP 1: Selection of Enumeration Area

(1) List of EA with number of HH by district for Census 2010 was obtained from the Directorate of Statistics. There are 1826 EAs in Timor-Leste. Out of these, 150 EAs were selected.

(2) The number of EAs to be selected from each district was based on their proportion in the country’s population as per Census 2010.

(3) The numbers of Households (HH) per EAs varied from 0 to more than 300. Therefore, probability proportion to size (PPS) was used.

(4) For each district, the EAs were arranged in ascending order of HH size.

(5) Sampling interval was obtained by dividing the total number of HH in the district by the number of EA to be selected from that district.

(6) A random number was generated between one and the sampling interval for that district, using tools available at random.org.

(7) The EA where that random number fell was the first EA to be selected.

(8) Subsequently, the sampling interval was added to the random number and the EA where this new number fell was selected. For the next number, the sampling interval was added to the number and so on, till the population of HH was exhausted or target number of EA achieved.

(9) This was done separately for each district.

(10) The final list was compiled and had 150 EAs. These are spread over about 125 sucos.

STEP 2. Selection of Households in an Enumeration Area

Listing the house numbers to be visited

(1) It was decided to use the 2010 HH size of each EA. Based on past experience, it was expected that the increase would be on an average about 4–5%.

(2) The list of households to be selected by enumerators was decided centrally.

(3) Sampling interval was calculated by dividing the total number of households in the EA by 18.

(4) The first HH number was selected randomly by reading the last two digits of a currency note. If the number represented by the two digits was more than 18, the last digit was taken into consideration. For each EA, a different currency note was used. This could also be done it by using the tool at random.org. or by draw of lots.

(5) The subsequent HH are identified by adding the sampling interval as was done for selection of EA.”

*Source: Timor-Leste STEPS Survey Report, [online] at http://www.who.int/entity/chp/steps/Timor-Leste_2014_STEPS_Report.pdf?ua=1*

**Togo: STEPS 2010**

“Those included in this survey are male or female subjects, living in urban or rural areas, aged 15 to 64 on the day of the survey, residing in the enumeration area for at least 6 months and having given their informed consent to participate in this study. [...] Three hundred clusters were randomly selected in a systematic draw with probability proportional to the size of the cluster (number of households) in the 4620 areas of enumeration of the DGSCN (General Directorate of Statistics and National Accounts) sampling frame. In order to obtain the 4,800 households at the rate of 1 individual / household, 16 households per cluster were randomly selected at the second stage of survey. In each of the selected households, one individual was selected as a survey participant via the Kish Method. A household was defined as the group of persons, who regularly share the main meal (regardless of their relationship). Households were not replaced in the event of a refusal or two unsuccessful visits to the eligible person selected by Kish's method. If the selected person was unwell or not present at the time of the interview, the investigators either tried to find a new appointment or searched for the respondent.”

*Source: Translated from WHO: The Final Report on the Togo STEPS Survey 2010. Available at: http://www.who.int/chp/steps/2010STEPS_Report_Togo_FR.pdf?ua=1.*

**Uganda STEPS 2014**

“Uganda has a total population of 34.9 million people, approximately 43% of which are adults aged 18 years or older [14]. The survey covered the whole country, and a three stage sampling design was used to select participants. The sampling procedure utilized the Uganda Bureau of Statistics (UBOS) master sampling frame of Enumeration Areas (EAs) that had just been demarcated throughout the country in preparation for the 2014 population and housing census. Each EA included 150–200 households. In the first stage, a random sample of 350 out of 78,950 EAs was selected with selection probability proportional to the size (PPS) of the number of households in the EAs. The EAs were stratified across the four regions of Uganda namely: Central, Eastern, Northern and Western region; and were selected with separate estimates for rural and urban areas. Urban areas were defined as EAs within government designated urban areas, or those within other geographic divisions with population density of more than 1000 per square kilometer.

After selecting the 350 EAs, trained teams of UBOS staff were dispatched throughout the country to list the households and their household heads within the 350 EAs. A household was defined as a group of individuals that usually shared meals together, and had a household head who usually made major decisions for the household. In the second stage of sampling, 14 households were randomly selected from the listed households in each of the sampled EAs.

Research Assistants (RA) that had received a five-day training on procedures and administration of the STEPs tool, enumerated eligible household members who were recorded in Personal Digital Assistants (PDA), which was then used to randomly select one subject for inclusion in the survey giving a total sample of 4900. Eligible subjects were household members aged 18 to 69 years, who had resided in the sampled households for at least six months preceding the date of interview.”

*Source: Guwatudde D, Mutungi G, Wesonga R, Kajjura R, Kasule H, Muwonge J, et al. (2015) The Epidemiology of Hypertension in Uganda: Findings from the National Non-Communicable Diseases Risk Factor Survey. PLoS ONE 10(9): e0138991. doi:10.1371/journal.pone.0138991.*
