# Supplementary material for: Health system performance for people with diabetes in 28 low- and middle-income countries: A cross-sectional study of nationally representative surveys
Source: PLoS Med. 2019 Mar 1;16(3):e1002751. doi: 10.1371/journal.pmed.1002751 (PMC6396901; doi:10.1371/journal.pmed.1002751)
Supplement: S4 Appendix — (DOCX) [file pmed.1002751.s004.docx]

# Appendix 4: Detailed methodology for household wealth index calculation

Across surveys, several different wealth indicators were measured including continuous income, income categories, income quintiles, an asset index, or a combination of these (see table below). In an effort to homogenize wealth in the pooled analysis, we constructed household wealth quintiles for each survey.

| **Wealth Measure** | **Country** |
| --- | --- |
| Asset index | Bangladesh, India, Indonesia, Kenya, Namibia, Nepal, South Africa |
| Continuous income | Bhutan, China, Mexico, Romania, Timor-Leste |
| Continuous income and quintiles | Benin, Comoros*, Georgia, Liberia*, Swaziland*, Togo*, Uganda*, Tanzania |
| Continuous income and categories | Guyana, Mongolia* |
| Income categories only | St. Vincent & the Grenadines |
| No wealth indicators assessed | Burkina Faso, Chile, Costa Rica, Fiji, Seychelles |
| *Quintiles were not used as they displayed large discrepancies with respect to continuous income range or could not be correctly identified | |

The construction of wealth quintiles depends on the given wealth indicator. Countries using an asset index surveyed a range of assets, dwelling characteristics, and further country-specific variables. Utilizing the standard DHS approach, we used principle component analysis to derive an asset index, from which we create unweighted wealth quintiles. Countries using an income-based measurement mainly followed the STEPS template questionnaire put forward by the WHO. In this, respondents were asked about the average earnings (taking the past year) of the household in a week, month, or year. In cases where this question was left unanswered, a pre-coded estimate of the households’ annual income was indicated. This pre-coded estimate was usually expressed as quintiles and sometimes as categories that were defined by the countries’ survey teams. Using both the pre-coded estimates as well as the continuous income, we again created unweighted wealth quintiles. In this, we assumed that national incomes follow a log-normal distribution and made use of the procedure put forward by Harttgen and Vollmer (2013) in combining income quintiles and categories. In seven cases, we dismissed pre-coded quintiles or income as they displayed very large discrepancies with respect to the continuous income range or could otherwise not be correctly identified. However, as the pre-coded estimates were typically only asked of respondents that had not indicated a continuous income, this led to only minor information losses.

Reference:

Harttgen, K., & Vollmer, S. (2013). Using an asset index to simulate household income. Economics Letters, 121(2), 257-262.
