# Supplementary material for: Health system performance for people with diabetes in 28 low- and middle-income countries: A cross-sectional study of nationally representative surveys
Source: PLoS Med. 2019 Mar 1;16(3):e1002751. doi: 10.1371/journal.pmed.1002751 (PMC6396901; doi:10.1371/journal.pmed.1002751)
Supplement: S5 Appendix — (DOCX) [file pmed.1002751.s005.docx]

# Appendix 5: Detailed methodology for diabetes biomarkers by country

| **Diabetes Biomarker** | **Country** | **Post Hoc Adjustment**^*^ |
| --- | --- | --- |
| *Point-of-care fasting capillary glucose* | | |
| HemoCue® Glucose 201 Analyzer (HemoCue, Brea, California, USA) | Bangladesh, Namibia, Tanzania | None |
| CardioCheck® PA (pts Diagnostics, Indianapolis, Indiana, USA) | Bhutan, Burkina Faso, Kenya, St. Vincent & The Grenadines, Swaziland, Timor-Leste, Uganda | None |
| Accutrend® Plus (Roche, Basel, Switzerland) | Benin, Chile, Guyana, Liberia, Mongolia, Togo | Multiplied by 1.11 |
| CONTOUR® (Ascensia Diabetes Care Holdings AG, Basel, Switzerland) | Seychelles | None |
| multiCare-in© (Biochemical Systems International, Arezzo, Italy) | Georgia | None |
| FreeStyle Optium H glucometer | India | Multiplied by 1.11 |
| Unknown | Comoros | None |
| *Laboratory-based Assessment of Fasting Plasma Glucose* | | |
| Hitachi 7600 modular chemistry analyzer (Hitachi, Tokyo, Japan) | China | N/A |
| SYNCHRON® System (Beckman Coulter, Inc., Miami, Florida, USA) | Costa Rica | N/A |
| Semi-automated analyzer in a mobile laboratory setting (Biolyzer 100, Analyticon Diagnostics, Germany) | Nepal | N/A |
| Cobas 6000 analyzer (Roche Diagnostics, Indianapolis, Indiana, USA) | Romania | N/A |
| *Glycated hemoglobin A1c (HbA1c)* | | |
| Dried blood spots using a DCA 2000+ analyzer (Siemens/Bayer, Munich, Germany) | Fiji | N/A |
| Dried blood spots using the Hemocue system | Indonesia | N/A |
| Point-of-care In2ItTM device by Bio-Rad | Mexico | N/A |
| Venous blood using automated high performance liquid chromatography | South Africa | N/A |
| ^*^Post hoc adjustment to convert from capillary to plasma equivalents. | | |
