# Supplementary material for: Health system performance for people with diabetes in 28 low- and middle-income countries: A cross-sectional study of nationally representative surveys
Source: PLoS Med. 2019 Mar 1;16(3):e1002751. doi: 10.1371/journal.pmed.1002751 (PMC6396901; doi:10.1371/journal.pmed.1002751)
Supplement: S6 Appendix — (DOCX) [file pmed.1002751.s006.docx]

# Appendix 6: Select questions from generic STEPS Instrument used in constructing the diabetes cascades of care

The generic STEPS questionnaire upon which most of the surveys included in this study was based is available on the WHO’s website: [*http://www.who.int/ncds/surveillance/steps/instrument/STEPS_Instrument_V3.2.pdf*](http://www.who.int/ncds/surveillance/steps/instrument/STEPS_Instrument_V3.2.pdf)

Select questions used to construct the diabetes cascades of care are included in the table below:

| Have you ever had your blood sugar measured by a doctor or other health worker? | Yes  No |
| --- | --- |
| Have you ever been told by a doctor or other health worker that you have raised blood sugar or diabetes? | Yes  No |
| In the past two weeks, have you taken any drugs (medication) for diabetes prescribed by a doctor or other health worker? | Yes  No |
| Are you currently taking insulin for diabetes prescribed by a doctor or other health worker? | Yes  No |
| During the past three years, has a doctor or other health worker advised you to do any of the following?  (RECORD FOR EACH) |  |
| Reduce fat in your diet | Yes  No |
| Start or do more physical activity | Yes  No |
| Maintain a healthy body weight or lose weight | Yes  No |

There was some variability across surveys in the specific questions asked including the recall period (e.g., “ever” versus “in the past 12 months” for tested). Country-specific surveys will be shared upon request.
