# Supplementary material for: Health system performance for people with diabetes in 28 low- and middle-income countries: A cross-sectional study of nationally representative surveys
Source: PLoS Med. 2019 Mar 1;16(3):e1002751. doi: 10.1371/journal.pmed.1002751 (PMC6396901; doi:10.1371/journal.pmed.1002751)
Supplement: S7 Appendix — (DOCX) [file pmed.1002751.s007.docx]

# Appendix 7: Percent (n) of participants with diabetes missing predictor variables, by country

| **Country** | **Sex** | | **Age** | | **Educational Attainment** | | **Household Wealth Quintile** | | **Body Mass Index Classification** | |
| --- | --- | --- | --- | --- | --- | --- | --- | --- | --- | --- |
|  | % | n | % | n | % | n | % | n | % | n |
| Bangladesh | 0 | 0 | 0 | 0 | 0 | 0 | 0 | 0 | - | - |
| Benin | 0 | 0 | 0 | 0 | 0 | 0 | 8.77 | 5 | 0 | 0 |
| Bhutan | 0 | 0 | 0 | 0 | 0 | 0 | 0 | 0 | 1.33 | 1 |
| Burkina Faso | 0 | 0 | 0 | 0 | 0 | 0 | - | - | 0 | 0 |
| Chile | 0 | 0 | 0 | 0 | 0.74 | 4 | - | - | 4.46 | 24 |
| China | 0 | 0 | 0 | 0 | 0.31 | 2 | 2.16 | 14 | 2.01 | 13 |
| Comoros | 0 | 0 | 0 | 0 | 0 | 0 | 44.90 | 44 | 3.06 | 3 |
| Costa Rica | 0 | 0 | 0 | 0 | 7.67 | 29 | - | - | 10.05 | 38 |
| Fiji | 0 | 0 | 0 | 0 | 2.58 | 15 | - | - | 2.41 | 14 |
| Georgia | 0 | 0 | 0 | 0 | 5.34 | 14 | 9.54 | 25 | 1.91 | 5 |
| Guyana | 0 | 0 | 0 | 0 | 1.55 | 2 | 16.28 | 21 | 1.55 | 2 |
| India | 0 | 0 | 0 | 0 | 0 | 0 | 0 | 0 | 19.96 | 6243 |
| Indonesia | 0 | 0 | 0 | 0 | 0.59 | 3 | 0 | 0 | 2.37 | 12 |
| Kenya | 0 | 0 | 0 | 0 | 0 | 0 | 0 | 0 | 3.74 | 4 |
| Liberia | 0 | 0 | 0 | 0 | 0.67 | 2 | 34.23 | 102 | 3.02 | 9 |
| Mexico | 0 | 0 | 0 | 0 | 4.66 | 127 | 69.15 | 1885 | 8.95 | 244 |
| Mongolia | 0 | 0 | 0 | 0 | 21.25 | 17 | 2.50 | 2 | 1.25 | 1 |
| Namibia | 0 | 0 | 0 | 0 | 0.46 | 1 | 0 | 0 | 1.83 | 4 |
| Nepal | 0 | 0 | 0 | 0 | 0 | 0 | 0 | 0 | 1.01 | 2 |
| Romania | 0 | 0 | 0 | 0 | 8.98 | 23 | 8.98 | 23 | 0 | 0 |
| Seychelles | 0 | 0 | 0 | 0 | - | - | - | - | 0 | 0 |
| South Africa | 0 | 0 | 0 | 0 | 14.80 | 87 | 11.05 | 65 | 6.29 | 37 |
| St. Vincent & The Grenadines | 0 | 0 | 0 | 0 | 0 | 0 | 29.31 | 34 | 0 | 0 |
| Swaziland | 0 | 0 | 0 | 0 | 10.65 | 18 | 38.46 | 65 | 14.79 | 25 |
| Tanzania | 0 | 0 | 0 | 0 | 1.41 | 2 | 14.08 | 20 | 0.70 | 1 |
| Timor-Leste | 0 | 0 | 0 | 0 | 3.13 | 2 | 25.00 | 16 | 0 | 0 |
| Togo | 0 | 0 | 0 | 0 | 0 | 0 | 8.89 | 8 | 0 | 0 |
| Uganda | 0 | 0 | 0 | 0 | 0 | 0 | 23.81 | 10 | 9.52 | 4 |
