# Supplementary material for: Health system performance for people with diabetes in 28 low- and middle-income countries: A cross-sectional study of nationally representative surveys
Source: PLoS Med. 2019 Mar 1;16(3):e1002751. doi: 10.1371/journal.pmed.1002751 (PMC6396901; doi:10.1371/journal.pmed.1002751)
Supplement: S8 Appendix — (DOCX) [file pmed.1002751.s008.docx]

# Appendix 8: Prevalence (95% confidence interval) of clinical diabetes by country

| Country | Number of Respondents with Diabetes | Raw | Accounting for Sampling Design |
| --- | --- | --- | --- |
| Bangladesh | 785 | 10.7 (10.0, 11.5) | 10.1 (9.3, 11.0) |
| Benin | 57 | 1.6 (1.2, 2.1) | 1.7 (1.1, 2.7) |
| Bhutan | 75 | 2.8 (2.2, 3.5) | 2.3 (1.6, 3.3) |
| Burkina Faso | 99 | 2.5 (2.0, 3.0) | 2.7 (2.1, 3.6) |
| Chile | 538 | 11.0 (10.2, 12.0) | 9.1 (7.8, 10.6) |
| China | 648 | 7.4 (6.9, 8.0) | 7.4 (6.9, 8.0) |
| Comoros | 98 | 4.3 (3.5, 5.2) | 4.3 (3.4, 5.3) |
| Costa Rica | 378 | 14.6 (13.2, 16.0) | 11.2 (8.8, 14.1) |
| Fiji | 581 | 43.2 (40.6, 45.9) | 43.2 (39.7, 46.8) |
| Georgia | 262 | 8.3 (7.4, 9.3) | 5.5 (4.7, 6.5) |
| Guyana | 129 | 15.7 (13.2, 18.3) | 13.3 (10.7, 16.3) |
| India | 31,272 | 4.2 (4.1, 4.2) | 5.7 (5.5, 5.9) |
| Indonesia | 506 | 7.8 (7.2, 8.5) | 7.0 (4.9, 10.0) |
| Kenya | 107 | 2.7 (2.2, 3.2) | 1.9 (1.3, 2.6) |
| Liberia | 298 | 13.7 (12.3, 15.2) | 13.7 (11.8, 15.7) |
| Mexico | 2,726 | 30.2 (29.2, 31.1) | 34.3 (31.9, 36.8) |
| Mongolia | 80 | 5.1 (4.1, 6.3) | 5.4 (4.1, 7.1) |
| Namibia | 218 | 6.7 (5.9, 7.6) | 6.1 (5.1, 7.3) |
| Nepal | 198 | 5.3 (4.6, 6.1) | 4.2 (3.4, 5.2) |
| Romania | 256 | 13.0 (11.5, 14.6) | 13.0 (11.6, 14.6) |
| Seychelles | 179 | 14.4 (12.5, 16.5) | 11.7 (10.0, 13.5) |
| South Africa | 588 | 12.7 (11.8, 13.7) | 10.3 (8.4, 12.5) |
| St. Vincent & the Grenadines | 116 | 11.8 (9.8, 13.9) | 8.7 (7.1, 10.7) |
| Swaziland | 169 | 6.0 (5.2, 7.0) | 5.0 (4.1, 6.0) |
| Tanzania | 142 | 3.0 (2.5, 3.5) | 2.7 (2.1, 3.5) |
| Timor-Leste | 64 | 2.7 (2.1, 3.5) | 2.7 (2.1, 3.5) |
| Togo | 90 | 2.6 (2.1, 3.2) | 2.5 (1.9, 3.2) |
| Uganda | 42 | 1.2 (0.9, 1.7) | 1.4 (1.0, 2.1) |
