# Supplementary material for: Health system performance for people with diabetes in 28 low- and middle-income countries: A cross-sectional study of nationally representative surveys
Source: PLoS Med. 2019 Mar 1;16(3):e1002751. doi: 10.1371/journal.pmed.1002751 (PMC6396901; doi:10.1371/journal.pmed.1002751)
Supplement: S9 Appendix — (DOCX) [file pmed.1002751.s009.docx]

# Appendix 9: Sensitivity analyses of diabetes prevalence and cascade performance assuming all participants who were missing data on fasting status were non-fasting

| Sensitivity analysis | Prevalence | Testing | Diagnosis | Treatment | Control |
| --- | --- | --- | --- | --- | --- |
| All participants who were missing data on fasting status were assumed to be non-fasting | 8.7  (8.1 – 9.5) | 63.8  (57.1 – 70.1) | 44.7  (40.6 – 48.9) | 38.7  (35.5 – 42.1) | 23.0  (21.1 – 25.1) |
| Excluding India | 8.9  (8.3 – 9.7) | 63.4  (56.7 – 69.6) | 44.7  (40.5 – 48.9) | 38.6  (35.3 – 42.1) | 23.0  (21.1 – 25.1) |
| Values are percent (95% confidence interval). All estimates account for sampling design. | | | | | |
