# Supplementary material for: Health system performance for people with diabetes in 28 low- and middle-income countries: A cross-sectional study of nationally representative surveys
Source: PLoS Med. 2019 Mar 1;16(3):e1002751. doi: 10.1371/journal.pmed.1002751 (PMC6396901; doi:10.1371/journal.pmed.1002751)
Supplement: S10 Appendix — (DOCX) [file pmed.1002751.s010.docx]

# Appendix 10: Cascade of care for diabetes (95% confidence interval) by country

Table shows the percent of the total diabetic population that self-reported reaching subsequent stages in the care process, conditional on having reached the previous stage.

| Country | Testing | Diagnosed | Treated | Controlled |
| --- | --- | --- | --- | --- |
| Bangladesh |  | 41.0 (36.6, 45.6) | 35.9 (31.7, 40.3)* | 27.6 (23.8, 31.7) |
| Benin | 35.2 (20.4, 53.6) | 32.0 (17.5, 51.1) | 32.0 (17.5, 51.1) | 21.7 (10.8, 38.9) |
| Bhutan | 45.7 (32.5, 59.5) | 36.5 (24.8, 50.2) | 36.2 (24.5, 49.9) | 26.5 (17.1, 38.7) |
| Burkina Faso | 17.0 (10.0, 27.3) | 8.3 (3.8, 17.2) | 7.3 (3.1, 16.2) | 6.9 (2.8, 15.8) |
| Chile | 79.5 (73.4, 84.5) | 63.6 (57.2, 69.7) | 47.8 (40.6, 55.0) | 32.6 (25.8, 40.3) |
| China |  | 35.8 (32.2, 39.6) | 34.3 (30.7, 38.0) | 25.9 (22.7, 29.4) |
| Comoros | 73.5 (64.0, 81.2) | 57.1 (48.4, 65.5) | 54.1 (44.9, 63.0) | 38.8 (30.7, 47.5) |
| Costa Rica | 84.6 (77.8, 89.5) | 74.0 (64.8, 81.5) | 73.8 (64.6, 81.3) | 55.4 (49.1, 61.5) |
| Fiji |  | 33.9 (29.1, 39.0) | 32.2 (27.6, 37.1) | 9.6 (7.1, 12.9) |
| Georgia | 79.3 (71.4, 85.4) | 66.6 (58.6, 73.6) | 57.2 (49.0, 65.1)* | 39.6 (32.2, 47.5) |
| Guyana | 84.8 (73.8, 91.7) | 67.4 (55.7, 77.3) | 65.0 (53.2, 75.3) | 40.6 (31.1, 50.7) |
| India |  | 28.2 (26.8, 29.6) | 27.0 (25.6, 28.4)* | 15.2 (14.1, 16.4) |
| Indonesia |  | 20.7 (14.8, 28.2) | 20.1 (14.4, 27.3) | 7.4 (4.0, 13.3) |
| Kenya | 54.1 (36.4, 70.8) | 29.2 (15.9, 47.3) | 27.7 (14.7, 45.9) | 18.4 (8.2, 36.5) |
| Liberia | 15.1 (10.8, 20.6) | 6.7 (4.5, 9.9) | 6.0 (3.9, 9.4) | 4.0 (2.3, 7.1) |
| Mexico |  | 43.2 (38.8, 47.6) | 39.0 (34.8, 43.4)* | 15.9 (13.2, 19.0) |
| Mongolia | 36.7 (23.2, 52.6) | 25.5 (13.6, 42.6) | 23.6 (12.5, 39.9) | 14.2 (6.6, 27.9) |
| Namibia | 58.7 (50.6, 66.4) | 43.1 (35.4, 51.2) | 42.6 (34.9, 50.6) | 28.6 (21.6, 36.7) |
| Nepal | 56.7 (46.7, 66.1) | 44.5 (35.3, 54.0) | 43.6 (34.5, 53.1) | 28.0 (21.0, 36.3) |
| Romania |  | 77.3 (71.8, 82.1) | 17.6 (13.4, 22.8) | 17.2 (13.0, 22.3) |
| Seychelles | 96.0 (91.2, 98.3) | 60.3 (52.2, 67.8) | 56.7 (48.7, 64.5) | 36.6 (29.4, 44.5) |
| South Africa | 64.4 (53.9, 73.8) | 44.2 (34.8, 54.0) | 40.1 (31.3, 49.5)* | 21.4 (15.3, 29.1) |
| St. Vincent & the Grenadines | 92.9 (85.0, 96.8) | 78.1 (66.5, 86.5) | 75.7 (65.7, 83.5) | 61.5 (51.3, 70.7) |
| Swaziland | 57.0 (43.8, 69.2) | 37.0 (25.4, 50.4) | 36.0 (24.5, 49.3) | 26.4 (16.9, 38.8) |
| Tanzania | 41.0 (27.1, 56.5) | 39.3 (25.6, 55.0) | 37.2 (23.6, 53.3) | 24.4 (12.0, 43.3) |
| Timor-Leste | 14.1 (7.4, 25.0) | 10.9 (5.2, 21.5) | 9.4 (4.2, 19.6) | 4.7 (1.5, 13.6) |
| Togo | 27.6 (18.1, 39.8) | 19.8 (11.7, 31.4) | 17.8 (10.3, 29.1) | 16.6 (9.3, 27.8) |
| Uganda | 37.6 (21.7, 56.7) | 29.5 (15.6, 48.7) | 29.5 (15.6, 48.7) | 20.8 (9.8, 38.8) |
| All estimates account for sampling design.  *Includes only medication. Questionnaire did not query lifestyle advice. | | | | |
