# Supplementary material for: Health system performance for people with diabetes in 28 low- and middle-income countries: A cross-sectional study of nationally representative surveys
Source: PLoS Med. 2019 Mar 1;16(3):e1002751. doi: 10.1371/journal.pmed.1002751 (PMC6396901; doi:10.1371/journal.pmed.1002751)
Supplement: S11 Appendix — (DOCX) [file pmed.1002751.s011.docx]

# Appendix 11: Univariable regression analyses assessing the relationship between socio-demographic characteristics and testing, treatment, and control of diabetes in 28 low- and middle-income countries

| Covariate | Testing^†^ | Treatment | Control |
| --- | --- | --- | --- |
| Sex |  |  |  |
| Male | REF | REF | REF |
| Female | 1.43 (1.13 – 1.80) | 1.21 (1.02 – 1.45) | 1.21 (0.96 – 1.53) |
| Age |  |  |  |
| 15-34 years | 0.53 (0.44 – 0.63) | 0.26 (0.20 – 0.34) | 0.32 (0.21 – 0.48) |
| 35-44 years | REF | REF | REF |
| 45-54 years | 1.46 (1.36 – 1.56) | 2.46 (1.96 – 3.09) | 2.33 (1.72 – 3.15) |
| ≥55 years | 1.85 (1.55 – 2.20) | 4.57 (3.55 – 5.89) | 4.78 (3.44 – 6.65) |
| Educational Attainment |  |  |  |
| No formal schooling | REF | REF | REF |
| Primary school | 1.24 (0.88 – 1.76) | 1.02 (0.71 – 1.47) | 0.97 (0.70 – 1.34) |
| Secondary school or above | 1.80 (1.17 – 2.77) | 0.82 (0.53 – 1.27) | 0.91 (0.57 – 1.44) |
| Household Wealth Quintile^*^ |  |  |  |
| 1 | REF | REF | REF |
| 2 | 1.05 (0.89 – 1.24) | 0.96 (0.75 – 1.23) | 1.00 (0.81 – 1.24) |
| 3 | 1.24 (0.93 – 1.65) | 0.91 (0.71 – 1.15) | 0.92 (0.68 – 1.25) |
| 4 | 1.43 (1.02 – 2.01) | 1.14 (0.76 – 1.71) | 1.13 (0.73 – 1.76) |
| 5 | 2.31 (1.40 – 3.81) | 1.45 (0.83 – 2.53) | 1.48 (0.77 – 2.86) |
| Body Mass Index Classification^‡^ |  |  |  |
| Underweight | 0.68 (0.56 – 0.82) | 0.42 (0.29 – 0.62) | 0.43 (0.28 – 0.67) |
| Normal weight | REF | REF | REF |
| Overweight | 1.94 (1.69 – 2.23) | 2.15 (1.69 – 2.73) | 2.37 (1.94 – 2.88) |
| Obesity | 3.19 (2.68 – 3.80) | 3.19 (2.10 – 4.85) | 3.66 (2.74 – 4.87) |
| * Does not include Burkina Faso, Chile, Costa Rica, Fiji, and Seychelles due to a lack of data on household wealth quintile.  ^‡^ Does not include Bangladesh, where height and weight data were not collected for men.  ^†^ Does not include Bangladesh, China, India, Indonesia, Mexico, and Romania because the questionnaires used in these countries did not specifically query whether or not respondents had ever had a blood glucose test. | | | |
