# Supplementary material for: Health system performance for people with diabetes in 28 low- and middle-income countries: A cross-sectional study of nationally representative surveys
Source: PLoS Med. 2019 Mar 1;16(3):e1002751. doi: 10.1371/journal.pmed.1002751 (PMC6396901; doi:10.1371/journal.pmed.1002751)
Supplement: S1 Checklist — (DOCX) [file pmed.1002751.s012.docx]

# STROBE Checklist

|  | **Item No** | **Recommendation** |
| --- | --- | --- |
| **Title and abstract** | 1 | (*a*) Indicate the study’s design with a commonly used term in the title or the abstract **The title includes this information.** |
|  |  | (*b*) Provide in the abstract an informative and balanced summary of what was done and what was found  **This information is included in the Abstract.** |
| **Introduction** | | |
| Background/rationale | 2 | Explain the scientific background and rationale for the investigation being reported  **This information is provided throughout the Introduction.** |
| Objectives | 3 | State specific objectives, including any prespecified hypotheses  **This information is provided in the final paragraph of the Introduction.** |
| **Methods** | | |
| Study design | 4 | Present key elements of study design early in the paper  **This information is provided in the Methods, in the subsection on Data Sources.** |
| Setting | 5 | Describe the setting, locations, and relevant dates, including periods of recruitment, exposure, follow-up, and data collection  **This information is provided in the Methods, in the subsection on Data Sources.** |
| Participants | 6 | (*a*) Give the eligibility criteria, and the sources and methods of selection of participants. Describe methods of follow-up  **This information is provided in the Methods, in the subsection on Data Sources.** |
|  |  | (*b*) For matched studies, give matching criteria and number of exposed and unexposed  **N/A** |
| Variables | 7 | Clearly define all outcomes, exposures, predictors, potential confounders, and effect modifiers. Give diagnostic criteria, if applicable  **This information is provided in the Methods, subsections on Diabetes Biomarkers, Definitions of Diabetes, and Constructing the Diabetes Care Cascade.** |
| Data sources/ measurement | 8* | For each variable of interest, give sources of data and details of methods of assessment (measurement). Describe comparability of assessment methods if there is more than one group  **This information is provided in the Methods, under the subsections on Data Sources, Diabetes Biomarkers, Definitions of Diabetes, and Constructing the Diabetes Care Cascade.** |
| Bias | 9 | Describe any efforts to address potential sources of bias  **This information is provided in the Methods section, under the subsection on Statistical Analyses.** |
| Study size | 10 | Explain how the study size was arrived at  **This information is provided in the Methods, under the subsection on Data Sources.** |
| Quantitative variables | 11 | Explain how quantitative variables were handled in the analyses. If applicable, describe which groupings were chosen and why  **This information is provided in the Methods, under the subsections on Data Sources, Diabetes Biomarkers, Definitions of Diabetes, and Constructing the Diabetes Care Cascade.** |
| Statistical methods | 12 | (*a*) Describe all statistical methods, including those used to control for confounding  **This information is provided in the Methods section, under the subsection on Statistical Analyses.** |
|  |  | (*b*) Describe any methods used to examine subgroups and interactions  **This information is provided in the Methods section, under the subsection on Statistical Analyses.** |
|  |  | (*c*) Explain how missing data were addressed  **This information is provided in the Appendix. This was a complete-case analysis due to low missingness (<20%) for covariates.** |
|  |  | (*d*) If applicable, explain how loss to follow-up was addressed  **N/A** |
|  |  | (*e*) Describe any sensitivity analyses  **This information is provided in the Methods, under the subsection on Statistical Analysis.** |
| **Results** | | |
| Participants | 13* | (a) Report numbers of individuals at each stage of study—eg numbers potentially eligible, examined for eligibility, confirmed eligible, included in the study, completing follow-up, and analysed  **A flow diagram is included in the Appendix.** |
|  |  | (b) Give reasons for non-participation at each stage  **A flow diagram is included in the Appendix.** |
|  |  | (c) Consider use of a flow diagram  **A flow diagram is included in the Appendix.** |
| Descriptive data | 14* | (a) Give characteristics of study participants (eg demographic, clinical, social) and information on exposures and potential confounders  **This information is provided in Table 1 and Table 2.** |
|  |  | (b) Indicate number of participants with missing data for each variable of interest **This information is provided in the Appendix.** |
|  |  | (c) Summarise follow-up time (eg, average and total amount)  **N/A** |
| Outcome data | 15* | Report numbers of outcome events or summary measures over time  **This information is provided in the Results and Table 2.** |
| Main results | 16 | (*a*) Give unadjusted estimates and, if applicable, confounder-adjusted estimates and their precision (eg, 95% confidence interval). Make clear which confounders were adjusted for and why they were included  **This information is provided in Table 3 and the Appendix.** |
|  |  | (*b*) Report category boundaries when continuous variables were categorized  **These are reported in Table 3.** |
|  |  | (*c*) If relevant, consider translating estimates of relative risk into absolute risk for a meaningful time period  **N/A** |
| Other analyses | 17 | Report other analyses done—eg analyses of subgroups and interactions, and sensitivity analyses  **These results are reported in the Appendix.** |
| **Discussion** | | |
| Key results | 18 | Summarise key results with reference to study objectives  **This information is provided in the Discussion.** |
| Limitations | 19 | Discuss limitations of the study, taking into account sources of potential bias or imprecision. Discuss both direction and magnitude of any potential bias  **This information is provided in the Discussion.** |
| Interpretation | 20 | Give a cautious overall interpretation of results considering objectives, limitations, multiplicity of analyses, results from similar studies, and other relevant evidence  **This information is provided in the Discussion.** |
| Generalisability | 21 | Discuss the generalisability (external validity) of the study results  **This information is provided in the Discussion.** |
| **Other information** | | |
| Funding | 22 | Give the source of funding and the role of the funders for the present study and, if applicable, for the original study on which the present article is based  **We have provided this information in the section titled “Funding”.** |

*Give information separately for exposed and unexposed groups.

**Note:** An Explanation and Elaboration article discusses each checklist item and gives methodological background and published examples of transparent reporting. The STROBE checklist is best used in conjunction with this article (freely available on the Web sites of PLoS Medicine at http://www.plosmedicine.org/, Annals of Internal Medicine at http://www.annals.org/, and Epidemiology at http://www.epidem.com/). Information on the STROBE Initiative is available at http://www.strobe-statement.org.
