# Supplementary material for: Health system performance for people with diabetes in 28 low- and middle-income countries: A cross-sectional study of nationally representative surveys
Source: PLoS Med. 2019 Mar 1;16(3):e1002751. doi: 10.1371/journal.pmed.1002751 (PMC6396901; doi:10.1371/journal.pmed.1002751)
Supplement: S1 Text — (DOCX) [file pmed.1002751.s013.docx]

# Text 1: Country-specific contact information regarding accessing data used in this study

Data included in this study are only publically available for 7 of the 28 countries. The links to where data can be downloaded (upon free registration) are:

Bangladesh: https://dhsprogram.com/data/dataset/Bangladesh_Standard-DHS_2011.cfm?flag=0

Chile: https://www.minsal.cl/estudios_encuestas_salud/

China: https://www.cpc.unc.edu/projects/china/data/datasets

India: https://dhsprogram.com/data/dataset/India_Standard-DHS_2015.cfm?flag=0

Indonesia: https://www.rand.org/labor/FLS/IFLS/access.html

Mexico: http://www.ennvih-mxfls.org/english/ennhiv-3.html

Namibia: https://dhsprogram.com/data/dataset/Namibia_Standard-DHS_2013.cfm?flag=0

For the remaining 21 countries, including Benin, Bhutan, Burkina Faso, Comoros, Costa Rica, Fiji, Georgia: Guyana, Kenya, Liberia, Mongolia, Nepal, Romania, Seychelles, South Africa, St. Vincent & the Grenadines, Swaziland, Tanzania, Timor-Leste, Togo, Uganda, please contact Sue Gilbert at sgilbert@hsph.harvard.edu

* For the member countries of the Caribbean Public Health Agency (CARPHA) - Guyana and St. Vincent and the Grenadines - data were shared through a Data Use Agreement signed with the Executive Director of CARPHA. The Senior Technical Officer for NCDs (Dr.Glennis Andall-Brereton) can be contacted, if necessary.
